# Ultrastructural Studies on a Model Tintinnid – *Schmidingerella meunieri* (Kofoid & Campbell, 1929) Agatha & Strüder‐Kypke, 2012 (Ciliophora). II. The Oral Apparatus

**DOI:** 10.1111/jeu.12795

**Published:** 2020-05-11

**Authors:** Michael S. Gruber, Birgit Weißenbacher, Sabine Agatha

**Affiliations:** ^1^ Department of Biosciences University of Salzburg 5020 Salzburg Austria

**Keywords:** Adoral membranelles, character evolution, cladistic analysis, endoral membrane, fibrillar structures, phylogeny

## Abstract

The ultrastructure of the oral apparatus is supposed to be significant for elucidating more recent common ancestry and might thus provide support for particular groupings of oligotrichean ciliates. The transmission electron microscopical study on mainly cryofixed *Schmidingerella meunieri* specimens provides the first detailed data for tintinnids and Oligotrichea in general. Ten new characters are included into the cladistic analysis. These features together with the very limited body of literature suggest that substantial changes in the oral ultrastructure correlate only with the formation of a circular adoral zone in choreotrichids. Despite homoplasious morphological and ontogenetic adaptations to the planktonic lifestyle in halteriid hypotrichs and oligotrichids, their oral apparatuses generally retain the plesiomorphic ultrastructure of the Perilemmaphora. The highly complex ultrastructure of the adoral zone is thus able to accomplish an extension in the zone's functionality without obvious changes; only the position of the adoral zone at the apical cell portion together with a globular to obconical cell shape are apparently crucial. Merely, minute apomorphies characterise the Oligotrichea and tintinnids, respectively. Tintinnids with derived somatic ciliary patterns possess distinct microtubular bundles connecting the oral apparatus with the myoneme in the peduncle.

VISIONARY researchers like Entz (1885), Daday (1887), and Fauré‐Fremiet (1908) propagated the importance of cell features for the tintinnid systematics. Nevertheless, the first comprehensive classifications by Kofoid and Campbell (1929, 1939) considered exclusively the characteristics of the loricae. Even, when cytological investigation techniques, such as protargol staining and electron microscopy, became available for gathering taxonomically and phylogenetically relevant characters, these methods were less frequently applied in tintinnids than in other ciliate groups (Gold 1979; Hedin 1976; Laval 1972; Laval‐Peuto 1975, 1976); still, the rather robust lorica was favoured in tintinnid taxonomy owing to its simpler collection and preservation.

In ciliates, the organisation of the oral ciliature was historically used to discriminate higher taxonomic ranks (Kahl 1930, 1932). Since it displays many homoplasies (Lynn 1981), the supposedly more conserved ultrastructure of the somatic kinetids was suggested for inferring relationships (Gerassimova and Seravin 1976; Lynn 1976). Nevertheless, the oral structures might be significant for elucidating more recent common ancestry. So, apomorphies related to the involvement of the adoral zone in locomotion of Oligotrichea, the formation of a circular adoral zone in choreotrichids, the jumping movements of several aloricate choreotrichids, or the development of the supposedly cumbersome lorica in tintinnids might emerge by comparing the few transmission electron microscopical studies in the hypotrich outgroup (Bąkowska and Jerka‐Dziadosz 1978; Fleury et al. 1985; Grain 1972; Grim 1972; Grimes 1972; de Puytorac et al. 1976; Torres et al. 1986), the oligotrichids, aloricate choreotrichids, and tintinnids (Bardele et al. 2018; Grim 1987; Hedin 1975, 1976; Laval 1971, 1972; Laval‐Peuto 1975, 1976; Laval‐Peuto et al. 1979; Sokolova and Gerassimova 1984; Wasik and Mikołajczyk 1991).

While the first part of our ultrastructural investigations on the model tintinnid *Schmidingerella meunieri* (Kofoid & Campbell, 1929) Agatha and Strüder‐Kypke, 2012 treated the somatic ciliature (Gruber et al. 2018b), the present second part focuses on the oral apparatus, i.e. the adoral zone of membranelles, the endoral membrane, and their associated fibres. It is the first detailed study in tintinnids and Oligotrichea in general.

## Materials and Methods

### Cultivation

Monoclonal cultures of the tintinnid originally designated as *Favella taraikaensis* and identified by SA as *S. meunieri* based on lorica features had been established on specimens sampled in the Northeast Pacific, i.e. at the coast of Washington State. Subsamples of these cultures were provided by Kelley Bright and Suzanne L. Strom from the Shannon Point Marine Centre, Western Washington University, USA. The specimens obtained in July 2011 and June 2016 were further cultivated in the laboratory in Salzburg with artificial sea water (salinity 33‰) plus a f/2 trace metal solution (Guillard 1975) at a 12 h:12 h light‐dark cycle and a temperature of 18 °C. The dinoflagellate *Heterocapsa triquetra* and the haptophyte *Isochrysis galbana* were used as food, but the cultures also contained a huge variety of further flagellates of unknown identity (SA own observ.).

### Fixation for transmission electron microscopy

Two fixation techniques were applied*,* namely, a chemical fixation and cryofixation.

#### Chemical fixation

The cells were picked with a finely drawn Pasteur pipette and were placed in block dishes containing a solution of 3% glutaraldehyde and 2% osmium tetroxide in 0.2 M cacodylate buffer (1,000 mOsm) at a ratio of 1:1. After 1 h in ice‐cooled block dishes, the specimens were washed three times with precooled 0.2 M cacodylate buffer for 10 min each and subsequently placed in precooled 4% glutaraldehyde for 1 h. Three washing steps with precooled 0.2 M cacodylate buffer for 10 min each followed. Then, the cells were put in ice‐cooled 2% osmium tetroxide for 1 h. Finally, the cells were washed again three times with precooled 0.2 M cacodylate buffer for 10 min each and stored in 70% ethanol at 4 °C overnight. The subsequent dehydration process on ice packs was done in block dishes with precooled ethanol at concentrations of 80%, 90%, and 96% for 15 min each, followed by three steps with 100% ethanol for 15 min each. Next, the specimens were washed three times for 10 min each in propylene oxide before embedding in EPON 812 (Serva Electrophoresis, Heidelberg, Germany). The embedding process comprised three steps, namely (i) a propylene oxide:EPON ratio of 3:1 for 1 h, (ii) a ratio of 1:1 for 2 h, and (iii) a ratio of 1:3 overnight in small aluminium dishes in a desiccator. Subsequently, polymerisation was performed by placing the samples in an incubator at 40 °C for 1 d and finally at 60 °C for 2 d.

#### Cryofixation

Since the method is described in detail in Gruber et al. (2018b), only the main steps are mentioned here. The live cells were transferred into sample holders with a minute amount of culture medium and placed into a Leica EM PACT high‐pressure freezer (Leica Mikrosysteme GmbH, Vienna, Austria) for cryofixation. The sample holders containing the fixed specimens were put in Eppendorf tubes filled with a precooled mixture of 2% osmium tetroxide and 0.05% uranyl acetate in acetone to perform freeze substitution in a Leica EM AFS (Vienna, Austria). Afterwards, the samples were rinsed in anhydrous acetone and washed in propylene oxide before being embedded in Agar Low Viscosity Resin (ALVR; Agar scientific, Stansted, UK). Next, the samples were placed in a desiccator and finally in an oven for polymerisation.

### Ultramicrotomy and electron microscopy

The polymerised samples were ultrathin‐sectioned (70 nm) with an ultramicrotome (Ultracut S, Reichert AG, Vienna, Austria). The investigations were conducted by means of a Zeiss EM 910 transmission electron microscope (Karl Zeiss AG, Oberkochen, Germany), and the micrographs were taken with a Sharp:Eye digital camera system (Tröndle), using the computer software ImageSP Viewer (SysProg & TRS, Moorenweis, Germany). The present data base on about 6,000 images from nine cryofixed specimens and eight chemically fixed cells.

### Computer aided 3D modelling

The 3D modelling of an adoral polykinetid and its internal connections was performed with the licensed computer software “SketchUp Pro” (www.sketchup.com), while the model of the peristomial rim with the collar polykinetids and the associated fibrillar structures was made with the freeware “Blender” (www.blender.org).

### Micrographs and measurements

The orientation of the micrographs is parallel to the longitudinal axis of the cell, if not stated otherwise. All micrographs show the cell in surface view, but the terms “right” and “left” refer to the perspective of the cell, the organelle, or the structure described. All measurements of the 3D structures in 2D sections are approximations owing to the scarcity of complete serial sections.

### Terminology

In the following, we suggest a uniform terminology for facilitating comparative ultrastructural studies in spirotrich ciliates.

#### Membranelles

**Figure 1:**
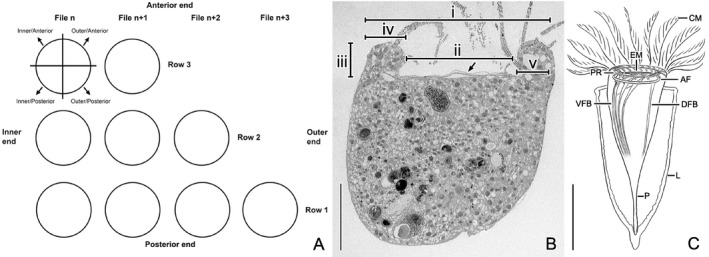
Scheme introducing the unified terminology concerning the adoral polykinetids (**A**), a longitudinal section of a *Schmidingerella meunieri* cell in the transmission electron microscope (**B**), and a scheme of a specimen displaying all relevant structures (**C**). (A) The collar polykinetids in choreotrichids consist of three rows and many files of basal bodies. They extend from the peristomial field (inner end) obliquely across the peristomial rim, terminating near the somatic cortex (outer end). The absence of kinetodesmal fibrils prevents a reliable triplet numbering; instead, the position of associated structures is described by pragmatically dividing the basal bodies into quarters. (B) Longitudinal section explaining the measurements of the peristomial rim, i.e. the roman numerals denote its outer diameter (i), its inner diameter (ii), its height (iii), the width of its apical portion (iv), and the width of its proximal portion (v). The arrow marks the endoral membrane. (C) Extended cell in its lorica. The ventral and dorsal fibre bundles apparently merge with the contractile myoneme (not shown) in the posterior cell portion. AF = adoral fibre; CM = collar membranelles; DFB = dorsal fibre bundle; EM = endoral membrane; L = lorica; P = peduncle; PR = preoral ring; VFB = ventral fibre bundles. Scale bars = 20 μm (B), 50 (C).

The adoral zones of membranelles are homologous in hypotrichs, oligotrichids, and choreotrichids. The terminology of the polykinetid structure with its basal body rows and files follows Lynn (1981, 1988), while the numbering of the rows is according to Bąkowska and Jerka‐Dziadosz (1978) (Fig. 1A).

**Figure 2:**
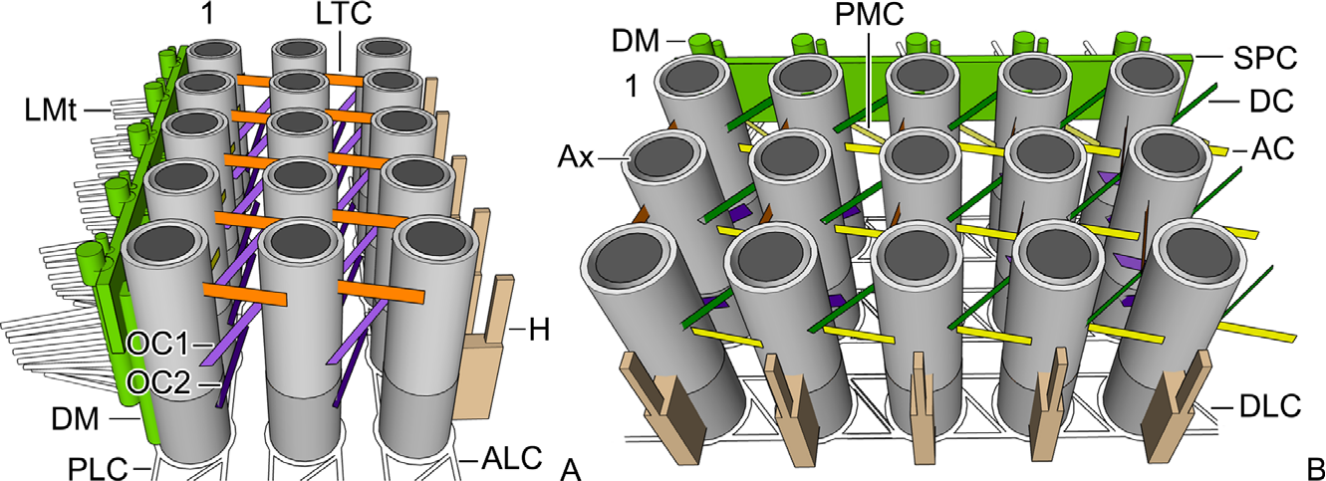
*Schmidingerella meunieri*, two aspects of a 3D reconstruction (SketchUp Pro; www.sketchup.com) of an adoral polykinetid with three rows and five files of basal bodies showing the very complex network of electron‐dense linking structures. (**A**) View on the outer end of the polykinetid with the posterior margin directed to the left. (**B**) View on the anterior margin of the polykinetid with the inner end directed to the right. Dark grey, cartwheel portions of basal bodies; light grey, basal body regions with core granules; 1 = basal body row 1; AC = anterior connections; ALC = anterior longitudinal connections; Ax = axosomes; DC = diagonal connections; DLC = diagonal longitudinal connections; DM = electron‐dense matter; H = hook‐shaped structures; LMt = lateral microtubules; LTC = left transverse connections; OC1 = oblique connections 1; OC2 = oblique connections 2; PLC = posterior longitudinal connections; PMC = postmembranellar connections; SPC = single posterior connection.

Since the early stages of stomatogenesis in Oligotrichea recapitulate the C‐shaped pattern and ventral placement of the adoral zone in the hypotrich outgroup, the orientation of the polykinetids in hypotrichs is used for unifying the terminology. Accordingly, the polykinetid's anterior margin extends along the last row of basal bodies and its posterior margin along the first row (Fig. 1A). In morphostatic choreotrichids, the polykinetids obtain an angle of about 45° to the course of the horizontally orientated circular adoral zone; hence, row 1 is orientated to the right and row 3 to the left. The inner ends of the polykinetids are adjacent to the peristomial field and the outer ends are close to the somatic cortex. Concerning the internally (between basal bodies of a polykinetid) and externally (between polykinetids) associated structures of the adoral polykinetids, the terminology is again according to Bąkowska and Jerka‐Dziadosz (1978) with a few exceptions. (i) The terms postciliary and transverse ribbons are not used as the absence of kinetodesmal fibrils in the ciliary structures prevents a reliable triplet numbering indispensable for inferring homology of the ribbons. Instead, the associated structures found in the present study are not named, but their positions are described by pragmatically dividing the basal bodies into quarters (Fig. 1A). (ii) The term “double posterior connection” is modified to “single posterior connection” (SPC) to describe the situation in *S. meunieri*. (iii) The term “diagonal longitudinal connections” (DLC) is introduced for links at cartwheel level extending between the inner‐posterior and the outer‐anterior quarters of adjacent basal bodies in each row (Fig. 2 and Fig. S3D, S5A, B).

The term “paramembranelle” defined by Lynn (2008) requires an emendation due to the new findings: *Paramembranelle* — specialised term for each of the several adoral membranelles characteristic of free‐living heterotrichs and spirotrichs; all basal bodies (kinetosomes) of the polykinetid are linked by similar‐appearing connectives; potential transverse microtubular ribbons are limited to the kinetosomes of the last (anteriormost) row of the polykinetid, but lack in tintinnids.

Additionally, the terminology of the circular structures in the peristomial rim of choreotrichids is unified. The adoral fibre was first described in iron‐haematoxylin‐stained cells of *Schmidingerella* sp. (reported as *Favella* sp.) and *Stenosemella nivalis* (reported as *Tintinnopsis nucula*) by Campbell (1926, 1927), who considered it part of the so‐called neuromotor apparatus. Although he probably confused the adoral fibre with the endoral membrane (Foissner and Wilbert 1979), Campbell mentioned a fibre system connecting all membranelles. The term “adoral fibre” is used sensu Laval (1972) but with a modification: The “adoral fibre” is a thick microtubular bundle formed by nematodesmata originating in the inner and outer portions of the collar polykinetid and the endoral membrane. The term “preoral ring” was probably introduced by Hedin (1976). It is here applied to the thinner and more apical microtubular bundle formed by nematodesmata originating in the middle portions of the collar polykinetids. The co‐occurrence of both rings was first described in protargol‐stained specimens of the tintinnid *Tintinnopsis everta* by Gruber et al. (2018a).

#### Endoral membrane

The terminology concerning the endoral membrane follows Grim (1987). The descriptive terms proximal/distal and right/left refer to the organelle.

### Cladistic analysis

The majority method was applied, which codes a polymorphic taxon as having the trait that is most common among the taxa considered (Wiens 2000). The present findings are preliminary as they are inferred from a very limited database, and taxon and character sampling might influence our hypotheses on taxon and character evolution.

## Results

The oral apparatus of *S. meunieri* occupies the apical cell portion and comprises about 17 collar membranelles, one buccal membranelle, and an endoral membrane (Fig. 1C). The following observations are mainly from common collar membranelles; yet, the single buccal membranelle and the elongated collar membranelles extending into the buccal cavity apparently do not differ in terms of basal body arrangement and internal connections. Exceptions concerning external connections are stated in the text.

### General morphology of oral apparatus

**Figure 3:**
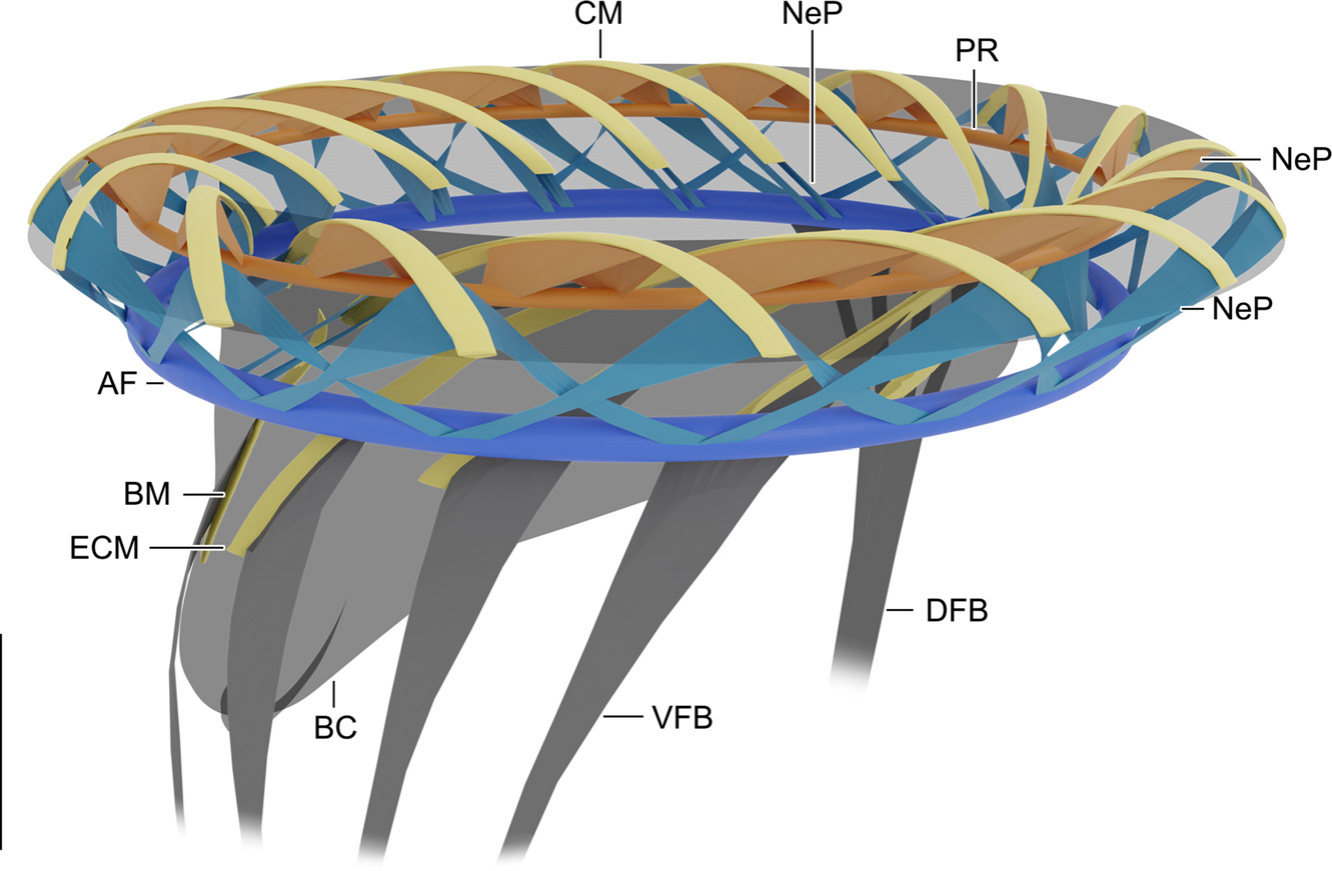
*Schmidingerella meunieri*, 3D reconstruction (Blender; www.blender.org) of the anterior cell portion displaying the peristomial rim with the adoral zone of membranelles and its associated microtubular bundles. Note that the lateral microtubules linking adjacent polykinetids are not shown for the sake of clarity. AF = adoral fibre; BC = buccal cavity; BM = buccal membranelle; CM = collar membranelles; DFB = dorsal fibre bundle; ECM = elongated collar membranelles; NeP = nematodesmata of adoral polykinetids (blue – from inner and outer portions; orange – from middle portions); PR = preoral ring; VFB = ventral fibre bundles. Scale bar = 10 μm.

The collar polykinetids extend obliquely across the peristomial rim, forming a closed circle with a contorted pattern around the peristomial field (Fig. 3 and Fig. S18); the oral apparatus remains perpendicular to the main cell axis in contracted cells (Fig. 1B, C).

**Figure 4:**
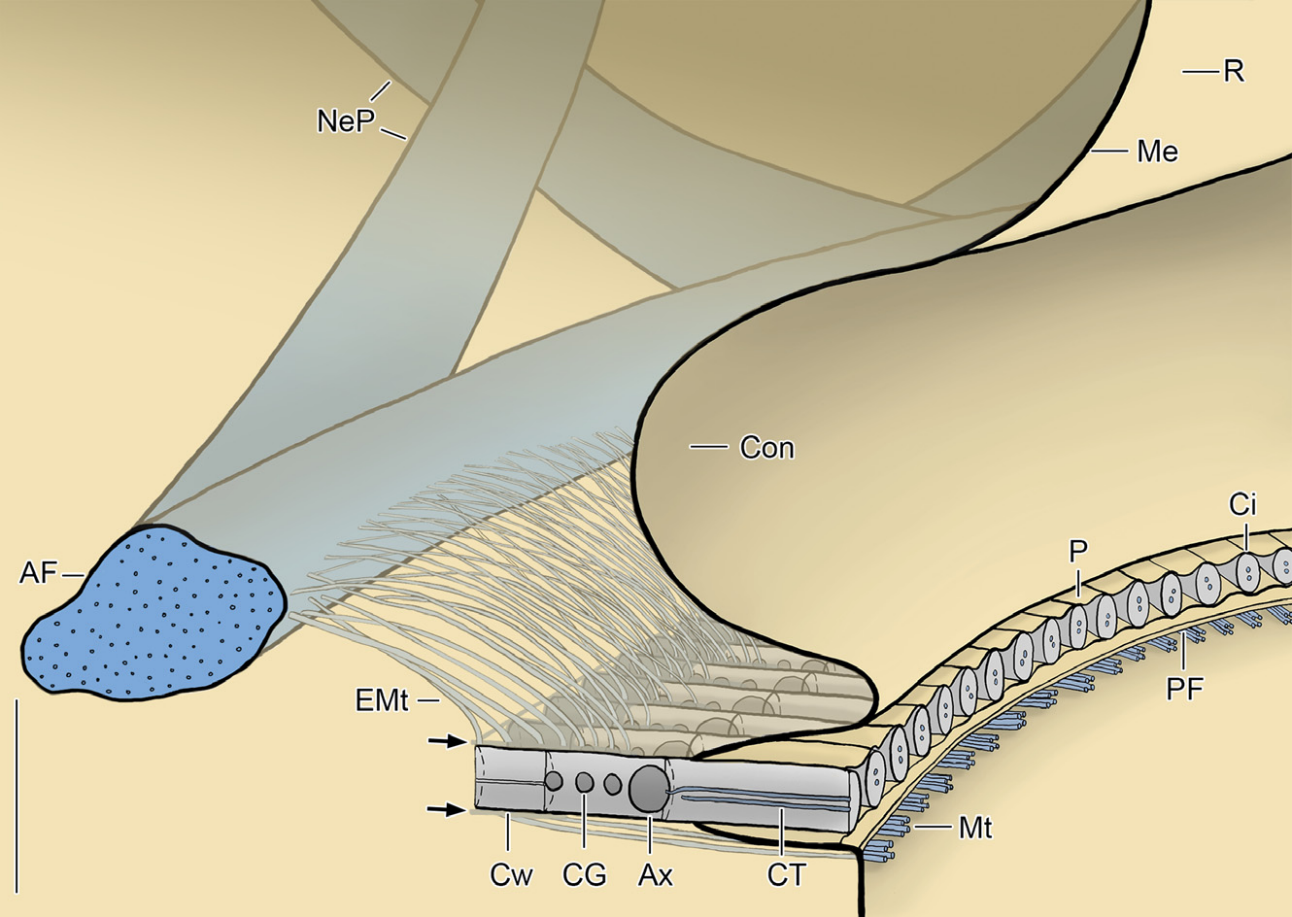
*Schmidingerella meunieri*, 3D reconstruction of semicircular endoral membrane extending parallel to the right half of the adoral fibre. In the main portion of the endoral, the microtubules extending from the right sides of the basal bodies to the adjacent adoral fibre measure < 1 μm, while they are distinctly longer (2–2.8 μm) and extend much more anteriorly in the distal portion of the endoral (cp. Fig. S13A, B). The presence of these associated microtubules is uncertain in the proximal portion. All endoral microtubules originate from electron‐dense material covering the basal bodies’ cartwheel and at least the right side in the core granule region. The basal bodies are connected by thin strips of electron‐dense matter on the right and left sides of the membrane (arrows). Note, that the membranous layers covering the endoral cilia are not shown for the sake of clarity. AF = adoral fibre; Ax = axosomes; CG = core granules; Ci = endoral cilia; Con = concavity of peristomial rim; CT = central microtubules; Cw = cartwheels; EMt = endoral microtubular bundles originating from the right sides of the basal bodies; Me = cell membrane; Mt = microtubular triplets or quadruplets originating from the left sides of the endoral basal bodies; NeP = nematodesmata of adoral polykinetids; P = perilemma; PF = peristomial field; R = projecting peristomial rim. Scale bar = 600 nm.

Two cryofixed and completely sectioned specimens with the membranelles slightly bent towards the peristomial field were morphometrically analysed. Their peristomial rims have (i) outer diameters of about 58.7 and 60.0 μm, (ii) inner diameters of about 30.1 and 38.6 μm, and (iii) heights of about 10.5 and 12.8 μm, respectively (Fig. 1B). The apical portions of the peristomial rims (iv) are about 12.5 and 10.9 μm wide on the left and ventral sides, while about 16.1 and 10.5 μm, respectively, on the right and dorsal sides, where they protrude over the peristomial fields and the proximal portions of the endoral cilia. In the latter region, the proximal rim portions (v) are only about 7.8 and 9.0 μm wide, respectively, due to the formation of an about 1–2 μm deep concavity, while they are about 9.9 and 10.5 μm wide on the left and ventral sides (Fig. 1B, 4 and Fig. S1A). In specimens having the membranelles bent outwards for feeding and swimming, the distances seem to be slightly enlarged. The diameter of the peristomial field is about 40.1 and 36.2 μm, respectively. The peristomial field is covered by membranous layers (Fig. S1A, S12C). It is invaginated in its ventral half, forming an oblique funnel (buccal cavity) with almost longitudinal walls on its right and ventral sides close to the proximal end of the endoral membrane and gradual slopes on its left and dorsal sides (Fig. 3 and Fig. S1C, D, S16A).

The collar membranelles are frayed distally (inferred from SEM images not shown). Their polykinetids consist of three rows of basal bodies having associated cilia up to 50 μm long. Four elongated collar polykinetids extend across the longitudinal ventral wall of the buccal cavity, which also bears one three‐rowed buccal polykinetid. The distances between adjacent polykinetids are highly variable (2.7–5.9 μm,$\bar{x}$ = 3.6 μm; *n* = 15) due to their oblique curvature across the peristomial rim whose outer circumference is larger than the inner one.

The endoral membrane consists of a single row of ciliated basal bodies (Fig. 4 and Fig. S1A–D, S12A–E, S13A, B, S14B, S16A, B). It commences in the dorsal portion of the peristomial field close to the dorsal kinety's anterior end and extends in a semicircle around the field's right half to the entrance of the buccal cavity (Fig. S1B, C). The endoral cilia extend horizontally across almost the entire peristomial field (Fig. 4 and Fig. S1A–D, S12C, S13A, B, S14B, S16A), except for the apparently shortened dorsal ones and the about 42 proximalmost cilia extending nearly to the buccal cavity's proximal end (Fig. S1C). The cell sections confirmed previous assumptions that the beating endoral cilia cause the conspicuous pumping movements in the peristomial area recognisable in live specimens (Fig. S16A). Up to two striae might be attached to a collar membranelle; the individual organelle measures about 1.4 × 1 μm in cross‐section (Fig. S2A, B). The intermembranellar ridges are indistinct and contain numerous granules (possibly extrusomes) and several vacuoles with fluffy content (Fig. S1D, S2B, S8C, S14C). Tentaculoids containing capsules and mucocysts insert in the ridges (Fig. S2C, S8C, D, S14C). The cell, except for the tips of the membranelles, is encompassed with perilemma.

### Ultrastructure of membranelles

The polykinetids are of the paramembranelle‐type with square‐packed basal bodies (de Puytorac and Grain 1976). All basal bodies are ciliated, but those in the inner and outer portions of the polykinetids might have associated condylocilia (= clavate cilia without central microtubules; Fig. S3A, B, S5C, S8B). The polykinetids are three‐rowed, except for their inner portions where the rows are successively shortened from posterior to anterior (Fig. S3A, C, S5C, S8C, D, S14A, S16B). In a common (not elongated) collar polykinetid, row 1 has about 90 basal bodies; about three condylocilia occur at its outer end, while none at its inner end. Row 2 has about 89 basal bodies; about three condylocilia occur at its outer end and two or three at its inner end. Row 3 has about 81 basal bodies; probably, more than three condylocilia occur at its outer end and one at its inner end. The numbers of basal bodies and condylocilia could not be determined in the elongated collar membranelles and the buccal membranelle.

The basal bodies within a polykinetid are not entirely equidistantly arranged, i.e. the basal bodies of a row are about 90 nm apart, while those of a file are about 110 nm apart. Many vacuoles occur between the rows at basal body level (observed in six specimens; Fig. S3D, S5A–D), while they are apparently absent within a row.

#### Polykinetidal basal bodies

The basal bodies of an adoral polykinetid invariably consist of nine microtubular triplets forming a cylinder about 590 nm high and about 220 nm across (Fig. S4A, B). The cartwheel is on average 220 nm high and occupies the proximal 37% of the basal body (Fig. S3B, S4B, S6A). At core granule level, the A‐tubules of adjacent triplets are linked with each other, while connections between A‐tubules and C‐tubules of adjacent triplets could rarely be identified (Fig. S4A). Terminal plates and axosomal plates were not recognisable. The lumen of the basal bodies contains three, rarely four, aligned electron‐dense core granules about 60 nm across (Fig. S3A, S4A, B, S5A, B, D, S6A). The axosome is globular, electron‐dense and about 120 nm across; thus, it almost fills the distal basal body lumen (Fig. S3A, S4A, B, S5D, S6A). Apparently, parasomal sacs are not associated with the polykinetids, and alveoli are absent in the apical cell region.

#### Internal connections

To facilitate understanding of the very complex, probably somewhat curved linkages between the basal bodies of an adoral polykinetid, a 3D scheme was developed (Fig. 2 and Fig. S17).

At cartwheel level, the basal bodies are surrounded by electron‐dense cuffs. Electron‐dense material also connects the basal bodies of each row, forming the posterior longitudinal connection (PLC) and the anterior longitudinal connection (ALC). Additionally, diagonal longitudinal connections (DLC) extend between the inner‐posterior and the outer‐anterior quarters of adjacent basal bodies in each row (Fig. 2 and Fig. S3D, S5A, B, S17).

Anterior connections (AC) link tangentially the middle anterior portions of the basal bodies in each row (Fig. 2 and Fig. S5D, S17). Directly proximal to the level of the axosome, left transverse connections (LTC) link tangentially the outer halves of the basal bodies in each file (Fig. 2 and Fig. S5C, D, S17). Diagonal connections (DC) are present in fragments. The fragments start in the core granule region at the outer‐posterior quarters of the basal bodies and extend obliquely to the inner‐anterior quarters of the basal bodies in file *n* + 1 of the same row (Fig. 2 and Fig. S4A, S5C, D, S6C, S17); between rows, the diagonal connections are indistinct. Oblique file connections 1 (OC1) commence at core granule level at the outer‐anterior quarters of the basal bodies in row *n* and extend to the inner‐posterior quarters just underneath the axosomes of the basal bodies in row *n *+ 1 of the same file (Fig. 2 and Fig. S4A, S5C, D, S17). The observations on the oblique file connections 2 (OC2) are fragmentary; the connections start somewhat more proximally than the oblique file connections 1 at the anterior halves of the basal bodies, but their courses could not be followed (Fig. 2 and Fig. S5A, S17). The basal bodies of row 1 are linked by postmembranellar connections (PMC) extending at core granule level between their outer‐posterior quarters and the thin longitudinal bulges along the basal bodies in files *n* + 1 (Fig. 2 and Fig. S5B, S17).

Electron‐dense material forms hook‐shaped structures extending longitudinally between the cartwheel and the distalmost core granule along the anterior sides of the basal bodies in row 3 (Fig. 2 and Fig. S5B, S6A–C, S7B, S8C, S17). Along the posterior margin of row 1, the single posterior connection (SPC) links the basal bodies tangentially at their middle levels (Fig. 2 and Fig. S3A, S5C, D, S8D, S17). This connection seems to be associated with paired bulges of electron‐dense matter (DM), i.e. one thicker outer and one thinner inner bulge per basal body extend longitudinally in the core granule region and perform short posterior curvatures with their distal portions (Fig. 2 and Fig. S4A, S5C, D, S17).

#### External fibrillar associates

Microtubules are associated with the polykinetids: (i) lateral microtubules (LMt) connect adjacent membranelles and (ii) nematodesmata (NeP) link the membranelles with two horizontally orientated circular fibre bundles in the peristomial rim; microtubules that might represent transverse or postciliary ribbons are not found.

Lateral microtubules (about nine or ten per basal body) originate from the electron‐dense matter (DM) along the basal bodies of row 1, except for the innermost and outermost basal bodies in which they are absent. They do not form bundles but are equidistantly spaced along the basal bodies and form more or less distinct fans perpendicular to the basal bodies’ main axes (Fig. 2A and Fig. S1A, S2B, S5A, B, S6A–D, S7A, B, S8C, S9A, S13A, S16B). They traverse the intermembranellar ridges, extending to the electron‐dense, hook‐shaped structures at the basal bodies in row 3 of the following polykinetid (Fig. S6B–D). The number of lateral microtubules reaching such a hook‐shaped structure is uncertain; once at least five microtubules were recognisable (Fig. S6B, D, S8C). Likewise, it is unclear whether lateral microtubules also commence at the anterior sides of the polykinetids.

Nematodesmata originate from most basal bodies; yet, their number per basal body could not be determined. Their fate differs depending on the position of the particular basal body in the polykinetid, i.e. the nematodesmata from basal bodies in the inner and outer collar polykinetid portions merge with the horizontally orientated, circular adoral fibre (Fig. 3 and Fig. S7A, S8B–D, S13A, S16A, S18), while those of basal bodies in the middle portions merge with the horizontally orientated, more apically located preoral ring (Fig. 3 and Fig. S7A, B, S9A, B, S18). A contribution of the buccal polykinetid's nematodesmata to the adoral fibre or preoral ring could not be verified.

In the collar polykinetids, the nematodesmata originating from the outermost basal bodies (about 16 as estimated in the protargol‐stained congener *Schmidingerella arcuata*; Fig. S15B) of rows 2 and 3 (rarely also of row 1) form two bundles, which curve centripetally in clockwise and anti‐clockwise directions (top view) through loose cytoplasm and merge with the adoral fibre about 11 μm apart from the polykinetid's outer end. Possibly, the nematodesmata of row 3 extend in clockwise and anti‐clockwise directions, while those of row 2 exclusively curve in anti‐clockwise direction (Fig. S7A, S8A, B, S13A). In their entirety, the outer nematodesmata generate a zigzagging pattern of argyrophilic bundles recognisable in lateral view (Fig. 3 and Fig. S7A, S8A, B, S10A, S15B, S18).

Concerning the inner portions of the common collar polykinetids, the nematodesmata originate from rows 1–3, although the sections might show only those of one row at a time (Fig. S8C, D, S16B). They form indistinct bundles, which extend posteriorly in clockwise direction before also merging with the adoral fibre (Fig. 3 and Fig. S8D, S18).

In the middle portions of the polykinetids, the nematodesmata originate from an undetermined number of basal bodies in rows 1–3 and extend singly or in bundles of up to four microtubules at angles of about 45° to the top of the peristomial rim in clockwise and anti‐clockwise directions, merging with the preoral ring after 2.3–4.7 μm (*n* = 11) (Fig. 3 and Fig. S7A, B, S9A, B, S18).

Both the adoral fibre and the preoral ring are roughly circular and almost horizontally orientated in the peristomial rim (Fig. 3 and Fig. S7A, B, S8B, S18). The adoral fibre is 5.0–5.3 μm (*n* = 3) more proximal than the preoral ring and extends near the inner margin of the peristomial rim. It is irregular in cross‐section and 770–930 nm (*n* = 3) wide. Its microtubules, whose number could not be ascertained, are linked by several thin connections (Fig. S16C). The preoral ring is about 3.6 μm (3.3–3.9 μm; *n* = 10) underneath the top of the peristomial rim and has an average thickness of about 596 nm (516–755 nm; *n* = 6).

Thick microtubular bundles extend posteriorly on the dorsal and ventral sides of the cell. The about 1 μm thick dorsal fibre bundle extends longitudinally close underneath the cell cortex. It consists of microtubular bundles originating in the adoral fibre and the distal portion of the endoral membrane (Fig. 3 and Fig. S10A, S13B, S15A, S18). It touches posteriorly the contractile myoneme, which is restricted to the posterior portion of cell proper and the peduncle (Fig. S10A, B). The ventral fibre bundles are formed by nematodesmata originating in the inner portions of the buccal polykinetid and of at least two, probably three elongated collar polykinetids. The ventral fibre bundles remain probably separate (Fig. 3 and Fig. S11A, B, S18) and might also terminate at the myoneme.

### Ultrastructure of endoral membrane

#### Endoral basal bodies

The basal bodies of the endoral membrane differ from those of the oral polykinetids in an electron‐dense region underneath the axosome (probably the axosomal plate; vs. not recognisable) and slightly smaller core granules (about 50 nm vs. 60 nm across) (Fig. 4 and Fig. S12A, D). The endoral has a stichomonad structure (see below). Its basal bodies are almost perpendicular to the main cell axis and all have associated cilia covered by perilemma and several membranous layers, probably also enclosing cytoplasm (Fig. 4 and Fig. S1A, S12C); yet, the number of basal bodies could not be ascertained. The distance between the basal bodies and the adoral fibre decreases from 2.0–2.8 μm in the distal portion of the endoral to < 1 μm in the remaining portion. For a very short distance, the proximal portions of the cilia are embedded into cytoplasm (Fig. S12B) and subsequently covered by a small epistomial lip (Fig. 4). Many small cytoplasmic inclusions or invaginations accompany the row of endoral basal bodies mainly on its left side, while they are absent between the basal bodies (Fig. S12A). Parasomal sacs were not found to be associated with the endoral membrane.

#### Internal connections

Thin strips of electron‐dense material link laterally the identically orientated endoral basal bodies between their cartwheels and first core granules (Fig. 4 and Fig. S12D); oblique connections are not recognisable.

#### External fibrillar associates

The endoral basal bodies have associated microtubular bundles on the left and right sides (EMt) originating from electron‐dense material; the number of microtubules per basal body could not be estimated. The microtubules commencing on the right sides from cartwheel to core granule level extend obliquely anteriorly before merging with the adoral fibre. They vary in length depending on the distance between the basal bodies and the adoral fibre. Their presence is uncertain in the endoral's proximal portion. The microtubules commencing at the proximal ends of the basal bodies’ left halves extend after performing a sharp curvature as triplets or quadruplets over about 800 nm parallel to the basal bodies and the endoral cilia directly underneath the peristomial field (Fig. 4 and Fig. S12A, B, D); their presence is uncertain in the endoral's proximal portion. In the distal portion of the endoral, further microtubules extend from the left sides of the basal bodies posteriorly, forming the dorsal fibre bundle together with microtubules originating in the adoral fibre (see above; Fig. S12E, S13A, B). In this region, also a distinct microtubular bundle seems to commence which curves in clockwise direction (top view; Fig. S12E, S13A, B, S15A); its length could not be determined. These microtubules might represent nematodesmata. Kinetodesmal fibrils were not visible.

### Buccal cavity and associated fibres

The eccentric buccal vertex is about 27 μm posteriorly to the top of the peristomial rim and about 8 μm underneath the ventrolateral cell surface. Towards the opening, the buccal cavity (see general morphology) comes by about 3 μm closer to the ventral cell surface.

In the left ventral portion of the adoral fibre, individual microtubules originate and extend over a short distance horizontally underneath the peristomial field (Fig. S14A, B) before they assemble regularly spaced bundles polygonal (triangular or trapezoidal) in cross‐section (Fig. S14C, D). The bundles with up to 58 microtubules each encompass the buccal cavity initially on the left, right, and dorsal sides, terminating near the buccal vertex (Fig. S1B, C). An accumulation of microtubules occurs posteriorly to the proximalmost elongated collar membranelle (Fig. S16D); yet, the origin of these microtubules could not be elucidated. Likewise, several microtubules with unknown course and vacuoles with fluffy content occur in the intermembranellar ridges in the buccal cavity (Fig. S16E).

## Discussion

With increasing speed, phylogenies based on gene sequence data are published. Particularly in tintinnids, this knowledge is indispensable as the main taxonomic feature complex, the lorica, shows homoplasies. Knowledge about the cell morphology would allow the establishment of a natural classification and of hypotheses on character evolution and adaptations; yet, the data are scarce. Although the oral ciliature displays homoplasies at higher taxonomic ranks, it is supposed to contain phylogenetic information for resolving/supporting lower ranks.

Thus, the present study aims to detect apomorphies by comparing the new findings on the tintinnid *S. meunieri* with the few detailed transmission electron microscopical studies on the oral apparatuses in hypotrichs (outgroup), oligotrichids, aloricate choreotrichids, and other tintinnids. These four taxa group in gene trees and are united in the Perilemmaphora (Berger 2008) based on the shared possession of a perilemma, a membranous layer surrounding the cell. The emerging derived characters might be related to the involvement of the adoral zone in the locomotion of the planktonic Oligotrichea, the formation of a circular adoral zone in choreotrichids, the jumping movements in several aloricate choreotrichids, or the development of the supposedly cumbersome lorica in tintinnids.

The monotypic genus *Lynnella* (Liu et al. 2011a) has a variable position in gene trees, i.e. it is sister to the oligotrichids or the choreotrichids depending on the phylogenetic analysis applied. Here, it is provisionally affiliated with the aloricate choreotrichids due to (i) its two longitudinal somatic ciliary rows, (ii) its derived kinetid structures (dikinetids with cilia only at the posterior basal bodies and monokinetids), (iii) its stomatogenesis in a subsurface pouch, and (iv) elongated proximalmost collar membranelles. Additionally, trichites and cortical platelets, as typical for oligotrichids, are absent.

### General aspects

#### Position, function, and shape of adoral zone of membranelles

In the majority of hypotrichs (outgroup), the adoral zone is mainly on the ventral side of the dorsoventrally flattened cell, and feeding is its only purpose as locomotion is performed by the ventral cirri. In Oligotrichea, the adoral zone is apical and used for both feeding and locomotion in the pelagial. The halteriid hypotrichs and the oligotrichids display homoplasies concerning the position and function of the adoral zone, which are interpreted as adaptations to the planktonic life style (Lynn and Kolisko 2017).

The adoral zone is C‐shaped in hypotrichs and oligotrichids, and the polykinetids are perpendicular to the course of the membranellar zone. In choreotrichids, the adoral zone is a closed circle, in which the membranelles form a contorted pattern on the peristomial rim; hence, their polykinetids are oblique to the course of the membranellar zone. The small ventral gaps in the adoral zones of the aloricate choreotrichids *Lynnella*,* Parastrombidium*, and *Parastrombidinopsis* probably evolved secondarily (Agatha and Strüder‐Kypke 2012, 2014; Kim et al. 2005; Liu et al. 2011a; Song et al. 2018a; Xu et al. 2007).

In the hypotrich outgroup (except for the halteriids), the adoral membranelles decrease gradually in width and the length of their cilia towards the cytostome; thus, a bipartition like in oligotrichids (and halteriid hypotrichs) with long and broad collar membranelles and small and short buccal membranelles is absent. In choreotrichids, the proximalmost collar membranelles have successively elongated polykinetids extending into the buccal cavity.

#### Number of basal body rows within a polykinetid

The adoral polykinetids of hypotrichs consist of four rows of basal bodies: two long rows (1 and 2) and two successively shortened rows (3 and 4); a few three‐rowed polykinetids might occur in the frontal zone portion. The halteriid hypotrichs are an exception; their collar polykinetids are three‐rowed and the buccal polykinetids are four‐rowed. Ultrastructural studies on Oligotrichea revealed three‐rowed polykinetids, except for the proximalmost polykinetid(s) which frequently appears to consist of only two rows (Agatha 2003a, 2004; Foissner et al. 1988; Grim 1987; Laval‐Peuto 1994; Liu et al. 2011b; Song and Bradbury 1998; Song et al. 2018b).

#### Number and position of undulating membranes

Hypotrichs have two undulating membranes arranged more or less parallel to the main cell axis (Berger 2008): the paroral membrane is on the outer wall of the buccal lip; the endoral membrane is on the bottom or right inner wall of the buccal cavity (Bąkowska and Jerka‐Dziadosz 1978). Oligotrichea have only one undulating membrane, namely, the endoral membrane, which differs in oligotrichids and choreotrichids concerning its orientation in relation to the cell's main axis: longitudinal on the inner wall of the buccal lip in oligotrichids (similar in *Lynnella semiglobulosa*!), while perpendicular and extending across the peristomial field in choreotrichids. The pumping movements of the peristomial area frequently described in tintinnids are apparently caused by the beating endoral membrane (Fig. S16A), which seems to be additionally covered by several membranous layers probably also enclosing cytoplasm (Sui et al. 2001); interestingly, the pumping movements have only been reported in one aloricate choreotrichid (Agatha 2003b).

### Ultrastructure

The polykinetids of the taxa discussed here are of the paramembranelle‐type with square‐packed basal bodies (de Puytorac and Grain 1976).

#### Internal connections of polykinetids

Only a few descriptions/images treat the internal connections of the polykinetids in tintinnids [*Cymatocylis*,* Cyttarocylis*,* Petalotricha*,* Ptychocylis*; Hedin (1976), Laval (1972), Laval‐Peuto (1975, 1976), Wasik and Mikołajczyk (1992)], aloricate choreotrichids [*Rimostrombidium lacustre*; reported as *Strobilidium velox* by Grim (1987)], oligotrichids [*Limnostrombidium*; Bardele et al. (2018)], and hypotrichs [including *Halteria grandinella*; Bąkowska and Jerka‐Dziadosz (1978), Fleury et al. (1985), Grain (1972), Grim (1972), Grimes (1972), de Puytorac et al. (1976), Torres et al. (1986)]. These restricted data suggest a high similarity of the internal connections among the abovementioned taxa (Table 1, 2). The hitherto most comprehensive description was published for the hypotrich *Paraurostyla weissei* (Bąkowska and Jerka‐Dziadosz 1978). It shows minute deviations from the pattern in *S. meunieri*: (i) double posterior connections vs. single posterior connections (SPC), (ii) the presence of anterior marginal connections, (iii) the presence of right transverse connections, and (iv) the absence of diagonal longitudinal connections (DLC).

**Table 1:** Character states and coding used for the cladistic analysis

	Characters	
	Apomorphic states	Plesiomorphic states
1	Adoral zone of membranelles mainly apical (coded 1)	Adoral zone of membranelles mainly ventral (coded 0)
2	Adoral zone of membranelles used for feeding and locomotion (coded 1)	Adoral zone of membranelles used for feeding only (coded 0)
3	Adoral zone of membranelles circular (coded 1)	Adoral zone of membranelles C‐shaped (coded 0)
4	Peristomial rim present; polykinetids oblique to zone's course (coded 1)	Peristomial rim absent; polykinetids perpendicular to zone's course (coded 0)
5^a^	Adoral zone bipartited, i.e. composed of buccal membranelles with small polykinetids and short cilia and collar membranelles with broad polykinetids and long cilia (coded 1) or with last collar polykinetids elongated extending into buccal cavity (coded 2)	Adoral zone not bipartited, i.e. polykinetids and cilia of adoral membranelles gradually decrease in size towards cytostome (coded 0)
6	Adoral polykinetids composed of three, rarely two rows (coded 1)	Majority of adoral polykinetids composed of four rows (coded 0)
7	Parasomal sacs absent (coded 1)	Parasomal sacs present (coded 0)
8	Terminal fibres absent (coded 1)	Terminal fibres present (coded 0)
9	Intermembranellar and postmembranellar fibres absent (coded 1)	Intermembranellar and postmembranellar fibres present (coded 0)
10	Adoral nematodesmata do not form submembranellar fibre (coded 1)	Adoral nematodesmata form submembranellar fibre (coded 0)
11	One undulating membrane, i.e. endoral membrane (coded 1)	Two undulating membranes, i.e. paroral and endoral membranes (coded 0)
12	Undulating membrane/s perpendicular to main cell axis (coded 1)	Undulating membrane/s parallel to main cell axis (coded 0)
13	Anti‐clockwise inclined links between endoral basal bodies absent (coded 1)	Anti‐clockwise inclined links between endoral basal bodies present (coded 0)
14	Lateral connections on one (coded 1) or both (coded 2) sides of endoral basal bodies	Lateral connections between endoral basal bodies absent (coded 0)
15	Dorsal fibre bundle present (coded 1)	Dorsal fibre bundle absent (coded 0)
16	Ventral fibre bundles present (coded 1)	Ventral fibre bundles absent (coded 0)

The coding is based on the comparison with the hypotrich outgroup. If not stated otherwise, the characters are ordered/additive (Wagner optimisation).

a Unordered character states.

**Table 2:** Distribution of character states in the hypotrich outgroup (Bąkowska and Jerka‐Dziadosz 1978) and the ingroup comprising the oligotrichids (Bardele et al. 2018), the paraphyletic aloricate choreotrichids (Grim 1987), and the tintinnids (mainly this study)

	Characters	Hypotrichs	Oligotrichids	Choreotrichids	
				Aloricates	Tintinnids
1	Position of adoral zone of membranelles	0^a^	1	1	1
2	Function of adoral zone of membranelles	0^a^	1	1	1
3	Shape of adoral zone of membranelles	0	0	1^b^	1
4	Peristomial rim with oblique polykinetids	0	0	1^c^	1
5^d^	Adoral zone bipartited	0^a^	1^e^	2	2
6	Basal body rows in polykinetids	0^a^	1	1	1
7	Parasomal sacs	0	1	1	1
8	Terminal fibres	0	1	1	1
9	Intermembranellar and postmembranellar fibres^f^	0	0	1	1
10	Submembranellar fibre	0	?	1	1
11	Undulating membranes	0	1^g^	1	1
12	Orientation of undulating membranes	0	0	1	1
13	Oblique links between endoral basal bodies	0	0	1	1
14	Lateral connections of endoral basal bodies	0	0	1	2
15	Dorsal fibre bundle	0	0	0	(1)^h^
16	Ventral fibre bundles	0	0	0	(1)^h^

For coding, see text and Table 1. If not stated otherwise, the characters are ordered/additive (Wagner optimisation).

?, Unknown character state.

a Majority rule applied, i.e. the halteriid hypotrichs which show several character states homoplasious to those of oligotrichids are excluded.

b Majority rule applied, i.e. the aloricate choreotrichid genera *Lynnella*,* Parastrombidium*, and *Parastrombidinopsis* with a supposedly secondary ventral gap are excluded.

c Majority rule applied, i.e. the monotypic genus *Lynnella* (provisionally assigned to the aloricate choreotrichids) with its longitudinally orientated polykinetids is excluded.

d Unordered character state.

e Majority rule applied, i.e. the oligotrichid genera *Williophrya* (without bipartition) and *Cyrtostrombidium* (without buccal membranelles) are excluded.

f Partially inferred from studies on protargol‐stained material.

Majority rule applied, i.e. the genus *Cyrtostrombidium* which apparently lacks undulating membranes is excluded.

Only in tintinnids with a *Schmidingerella*‐like or most complex somatic ciliary patterns.

Parasomal sacs are associated with the adoral polykinetids in hypotrichs (including *Halteria*) (Bąkowska and Jerka‐Dziadosz 1978; Grain 1972; Torres et al. 1986), while they are apparently absent in Oligotrichea.

#### External fibrillar associates of polykinetids

The adoral membranelles have associated numerous microtubules (Table 1, 2), which might be revealed by protargol staining when they form thick bundles.

Thick argyrophilic intermembranellar fibres composed of postciliary and transverse microtubules extend between and parallel to the polykinetids of hypotrichs (including *Halteria*) and merge near the polykinetid's outer ends to form the postmembranellar fibre, which reaches the cytostome (Bąkowska and Jerka‐Dziadosz 1978; Grain 1972). The scarce data from transmission electron microscopy and stained material of oligotrichids suggest postciliary and transverse ribbons contributing to the formation of intermembranellar and postmembranellar fibres in the collar region of the adoral zone (e.g. Agatha 2003a; Bardele et al. 2018; Song et al. 2000; Xu et al. 2007); regarding the buccal zone portion, the situation is uncertain. In aloricate choreotrichids, intermembranellar and postmembranellar fibres were neither illustrated nor described, although Grim (1987) reports transverse microtubules. In tintinnids, intermembranellar and postmembranellar fibres also do not occur due to the absence of postciliary and transverse microtubules (this study; Laval‐Peuto and Brownlee 1986). The transverse bundles mentioned and illustrated by Hedin (1976) are probably misidentified.

Lateral microtubules were first mentioned by Laval (1972) in tintinnids. They occur also in aloricate choreotrichids, oligotrichids, and hypotrichs. In hypotrichs and tintinnids, it is apparently variable whether lateral microtubules originate only from the anterior side, only from the posterior side, or from both sides of the polykinetids [e.g. this study, Bąkowska and Jerka‐Dziadosz (1978), de Puytorac et al. (1976), Laval (1972), Laval‐Peuto (1975, 1976), Torres et al. (1986)]. Aloricate choreotrichids possess lateral microtubules at least at row 1 (Grim 1987). Transmission electron microscopical data on oligotrichids are restricted to *Limnostrombidium viride*, which apparently has lateral microtubules merely associated with row 3 (Bardele et al. 2018); however, variability similar to that in hypotrichs and tintinnids cannot be excluded.

Terminal fibres originating from the innermost file of the polykinetids in hypotrichs extend towards the peristome and the cytostome (Bąkowska and Jerka‐Dziadosz 1978); ultrastructural studies suggest that they are absent in Oligotrichea.

Nematodesmata associated with the polykinetids were revealed by transmission electron microscopy in hypotrichs and choreotrichids. In at least some hypotrichs, the nematodesmata originating in the frontal/collar polykinetids form a zigzagging pattern and merge with one or possibly two bundles, while those of the ventral/lapel polykinetids run posteriorly underneath the adoral zone and form the frequently reported tuft‐shaped submembranellar fibre (Bąkowska and Jerka‐Dziadosz 1978; Kim et al. 2014). Interestingly, the protargol‐stained halteriid *Meseres* has the zigzagging pattern apparently associated only with the buccal polykinetids (Petz and Foissner 1992). In choreotrichids, the preliminary data suggest that the nematodesmata do not form a submembranellar fibre. Instead, those commencing in the outer portions of the collar polykinetids generate with thick argyrophilic bundles a zigzagging pattern [Fig. S15; this study; Grim (1987), Gruber et al. (2018a), Jiang et al. (2012), Lynn and Montagnes (1988)]; deviating findings based on only stained material require verification (Krainer 1995; Xu et al. 2007). The same is true for the supposedly aloricate choreotrichid *Lynnella semiglobulosa* (Liu et al. 2011a), whose pattern in protargol‐stained cells deviates from that in hypotrichs and choreotrichids. The presence of nematodesmata in oligotrichid polykinetids lacks ultrastructural confirmation; yet, in some protargol‐stained specimens, certain polykinetids seem to have associated zigzagging structures (Krainer 1991; Song et al. 2015a,b, 2020).

The nematodesmata bundles that originate in the inner portions of the polykinetids and merge with the adoral fibre are more indistinct. They are described here for the first time by means of transmission electron microscopy (Fig. S8C, D, S16C) and have previously been reported only once from protargol‐stained material (Gruber et al. 2018a). The same is true for the second, apically situated preoral ring, which is formed by the nematodesmata originating from the polykinetids’ middle portions (this study; Gruber et al. 2018a). In the aloricate choreotrichid *R. lacustre*, a structure corresponding to the adoral fibre is described in the text, while two transmission electron micrographs highly likely display the preoral ring in close vicinity to the adoral zone (Grim 1987).

During stomatogenesis of choreotrichids, the inverted C‐shaped oral primordium forms a closed circle of collar membranelles in middle dividers. Simultaneously, the adoral fibre and probably also the preoral ring form two circular (ventrally closed) structures as inferred from own observations on a protargol‐stained congener of the tintinnid species studied here (*S. arcuata*; MSG own observ.).

The previous findings indicate that the submembranellar fibres in hypotrichs are homologous to the adoral fibres and preoral rings in choreotrichids as all structures are formed by nematodesmata of the adoral polykinetids.

For the first time, microtubular bundles with a polygonal cross‐section and extending from the adoral fibre underneath the peristomial field to the buccal vertex have been described in tintinnids and choreotrichids in general. Possibly, the occurrence of these bundles correlates with the presence of the ring‐shaped adoral fibre; yet, more data are required for verification.

A special feature of tintinnids with *Schmidingerella*‐like or most complex somatic ciliary patterns seems to be conspicuous argyrophilic dorsal and ventral fibre bundles. The dorsal fibre bundle is composed of microtubules originating in the adoral fibre and the distal portion of the endoral membrane. The ventral bundles consist of nematodesmata from the inner portions of the buccal polykinetid and the elongated collar polykinetids; yet, their first occurrence in the tintinnid evolution is unknown. In *S. meunieri*, both bundles are in contact with the myoneme of the contractile peduncle as shown by transmission electron microscopy for the dorsal one (Fig. 3 and Fig. S10A, B) and by protargol‐stained cells for the ventral ones (not shown; MSG own observ.). The present investigation is the first ultrastructural study on these bundles and supports previous findings from protargol‐stained tintinnids [MSG own observations on the congener *S. arcuata*, Agatha (2008), Agatha and Riedel‐Lorjé (2006), Agatha and Tsai (2008), Gruber et al. (2018a), Jiang et al. (2012)]. The literature survey indicates some variability in the arrangement of the ventral bundles, i.e. the nematodesmata originating in the buccal membranelle and the elongated collar membranelles fuse to various degrees, forming 2–5 bundles.

#### Internal connections of endoral membrane

The endoral basal bodies of hypotrichs and oligotrichids are exclusively linked by anti‐clockwise inclined, electron‐dense connections (Table 1, 2; Bąkowska and Jerka‐Dziadosz 1978; Bardele et al. 2018). Choreotrichids apparently do not possess anti‐clockwise inclined connections. In aloricate taxa, a thin strip of electron‐dense material connects the right sides of the identically orientated basal bodies (Grim 1987), while another strip connects also the left sides in tintinnids as shown here for the first time.

#### External fibrillar associates of endoral membrane

In hypotrichs (incl. *Halteria*) and Oligotrichea [e.g. this study, Bardele et al. (2018), Grain (1972), Grim (1972, 1987), Laval‐Peuto and Brownlee (1986)], microtubules originate on the right (perhaps “postciliary microtubules”) and left sides (perhaps “transverse microtubules”) of the endoral basal bodies (Table 1, 2). Although the course and arrangement of these microtubules vary between the taxa, the data are insufficient for further analyses. For the first time, the contributions of endoral microtubules to the formation of the adoral fibre and the dorsal fibre bundle are demonstrated.

Nematodesmata originate from the endoral basal bodies in all taxa discussed here. The hypotrich nematodesmata diverge in two directions: they join (i) those of the paroral membrane and (ii) the submembranellar fibre [Bąkowska and Jerka‐Dziadosz (1978)]. In oligotrichids, they apparently extend exclusively through the base of the buccal lip towards the cytostome (Bardele et al. 2018). Data on aloricate choreotrichids are also sparse and describe the nematodesmata to extend into the cytoplasm (Grim 1987). The microtubules underpinning the peristomial field in tintinnids were identified as nematodesmata by Laval‐Peuto (1994); however, in *S. meunieri*, these microtubular triplets or quadruplets originate from the left sides of the endoral basal bodies. Microtubules that might represent nematodesmata in *S. meunieri* were only found to be associated with the distal portion of the endoral (Fig. S12E, S13A, B). They form a bundle which probably curves in clockwise direction anteriorly into the peristomial rim as inferred from protargol‐stained material of the congener *S. arcuata* (Fig. S15A).

### Phylogenetic inference

**Figure 5:**
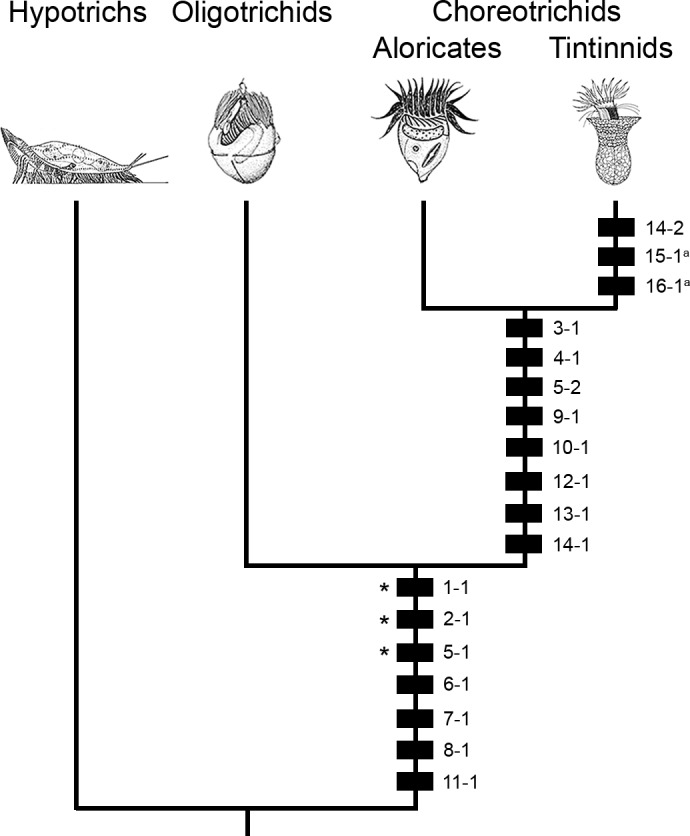
Phylogeny of Oligotrichea and the hypotrich outgroup. The apomorphies (black squares with numbers referring to the characters and their states listed in Table 1, 2) are mapped on the gene tree. The last common ancestor of the hypotrichs and Oligotrichea is supposed to possess (i) a ventrally located C‐shaped adoral zone of membranelles used for feeding only, (ii) generally four‐rowed membranelles decreasing gradually in size towards the cytostome, (iii) postciliary and transverse microtubular ribbons, which originate in the polykinetids and form intermembranellar fibres merging with the postmembranellar fibre, (iv) submembranellar fibres composed of nematodesmata of the adoral polykinetids, (v) terminal fibres and parasomal sacs associated with the adoral polykinetids, (vi) two undulating membranes, and (vii) anti‐clockwise inclined links between the endoral basal bodies, which have microtubular bundles on both sides. Homoplasies with the halteriid hypotrichs are marked by asterisks. ^a^Features possibly restricted to tintinnids with *Schmidingerella*‐like and most complex somatic ciliary patterns. Illustrations: hypotrich from Deitmer et al. (1984); oligotrichid from Montagnes (1996); aloricate choreotrichid from Petz and Foissner (1992); tintinnid from Gruber et al. (2018b).

The previous cladistic studies by Agatha and Strüder‐Kypke (2007, 2014) had a resolution on species and genus level, while the present analysis considers only higher ranks, namely, the hypotrich outgroup, the oligotrichids, the (nonmonophyletic) aloricate choreotrichids and the tintinnids because of the scarce data. Accordingly, exceptional species and genera are neglected, following the majority rule (Table 1, 2) (Wiens 2000). That applies also to the halteriids, which have recently been assigned to the hypotrichs and display homoplasious characters with the oligotrichids (Lynn and Kolisko 2017), i.e. the position (character 1), function (character 2), and bipartition (character 5) of the adoral zone. Ten characters are new, namely, characters 4, 7–10, and 12–16; further new characters emerged, but the current state of knowledge is too meagre for their cladistic analyses (see below). The character distribution among the abovementioned taxa has already been discussed in the preceding comparison (Table 2). We restrict our analyses to the Perilemmaphora. The characteristics of their last common ancestor are reconstructed by considering the euplotid sistergroup (Fig. 5). The hypotrichs serve as outgroup for mapping the apomorphies provided by the oral ciliature on a tree topology inferred from analyses of various marker genes (Fig. 5). Pragmatically, all character states found in the typical hypotrichs are regarded as plesiomorphic. However, the direction of transformation in the characters 7–10 might change in the future, when the states are known in euplotids.

Although they are supposed to be phylogenetically relevant, the following characters are not cladistically analysed here because of insufficient data; further investigations are required for detecting synapomorphies.

#### Internal connections of adoral polykinetids

The ultrastructural data available suggest a high similarity of the internal connections between the basal bodies among the taxa discussed. Nevertheless, minute deviations like those between a well‐studied hypotrich and the tintinnid *S. meunieri* might be found.

#### Lateral microtubules of polykinetids

These structures apparently occur in all taxa of Perilemmaphora. Their origin at the anterior, at the posterior, or at both marginal basal body rows might be a suitable character.

#### Nematodesmata of polykinetids

In the present analysis, only the presence of a submembranellar fibre is included as a character; however, stained cells suggest that the nematodesmata originating in different portions of the adoral zone might create different bundle patterns.

#### External fibrillar associates of endoral membrane

The preliminary findings indicate that the nematodesmata and the microtubules laterally associated with the endoral basal bodies extend in various directions in the taxa discussed here.

#### Buccal seal

Homology between the buccal seal, as described based on scanning electron microscopy in hypotrichs by Foissner and Al‐Rasheid (2006), and the membranous sheets covering the endoral membrane in transmission electron microscopical sections of oligotrichids and choreotrichids is unknown.

#### Pharyngeal fibres

This fibre system is known from various protargol studies on all taxa discussed here. However, the origin of these fibres could not be detected in the present and previous studies.

#### Microtubular bundles with polygonal cross‐sections

It is the first time that these bundles are described for Perilemmaphora. Possibly, they extend beyond the buccal vertex obliquely posteriorly into the cytoplasm, forming the pharyngeal fibre bundles which are usually visible in protargol‐stained material (see above).

### Adaptations

The first detailed data on a tintinnid presented here, namely, on *Schmidingerella meunieri*, allow a preliminary evolutionary reconstruction of the oligotrichean oral apparatus by comparison with the hypotrich outgroup (Fig. 5 and Table 1, 2).

Contrary to the expectations, the restricted data do not indicate that changes (i) in the cell shape from dorsoventrally flattened to globular, (ii) in the position of the adoral zone from the ventral to the apical cell portion, and (iii) in the function from feeding‐only to feeding‐and‐locomotion had any influence on the ultrastructure of the oral apparatus. This conclusion is based on the few observations on oligotrichids displaying the same plesiomorphic features as the benthic hypotrichs and the planktonic halteriid hypotrichs, with which they share several homoplasies (see above). Hence, the plesiomorphic ultrastructural features of the adoral zone apparently allow not only the generation of water currents for upstream filtration, but also a switch to forward and backward propulsion and a modulation of swimming patterns (Fenchel and Jonsson 1988). The apical position of the adoral zone in combination with a globular or obconical cell shape seems to be the sole character crucial for these additional functions relevant for the planktonic life style. Adaptations of the oral apparatus to the jumping movements in several aloricate choreotrichids or to the supposedly cumbersome lorica in tintinnids could not be discovered in the very limited body of literature.

Only when the adoral zone became circular in choreotrichids, apomorphies likely occurred: (i) the membranelles became obliquely orientated on a peristomial rim (Character 4); (ii) the proximal collar membranelles elongated into the buccal cavity (Character 5‐2); (iii) the intermembranellar and postmembranellar fibres disappeared (Character 9); and (iv) the tuft‐shaped submembranellar fibre was entirely replaced by two circular structures, the adoral fibre and the preoral ring (Character 10). In the last common ancestor of the choreotrichids, the endoral membrane also changed its orientation from parallel to perpendicular to the cell's main axis (Character 12). Simultaneously, the oblique connections between the endoral basal bodies disappeared and lateral connections evolved on one side in aloricate choreotrichids and finally on both sides in tintinnids (Characters 13 and 14). The pumping movements of the peristomial field caused by the beating endoral cilia are conspicuous in (all?) tintinnids, while it was reported only from a single aloricate choreotrichid (Agatha 2003b).

The connections of the dorsal and ventral fibre bundles with the myoneme in the contractile peduncle of *S. meunieri* (this study) suggest an involvement in the cell's retraction into the lorica. The comparison with tintinnids apparently lacking such bundles demonstrates, however, that the retraction is independent from their presence (see ‘External fibrillar associates of polykinetids’) and the intensity of the peduncle's contractility (own observ.). The first occurrence of the bundles in the tintinnid evolution is currently unknown. Likewise, it is uncertain whether the microtubular connections between the endoral and the adoral fibre as well as the polygonal microtubular bundles which originate in the adoral fibre and underpin the peristomial field were already present in the last common choreotrichid ancestor as these structures are described for the first time in the present study.

### Outlook

Generally, fibrillar structures associated with the ciliature increase the stability of the cell, independent of whether it concerns the somatic or the oral cell cortex. At the current, very restricted state of knowledge, however, no patterns emerged, i.e. the presence and/or arrangement of the oral fibrillar associates do not display a correlation with the life style, the possession of a lorica, or the peculiarities of several aloricate choreotrichids, viz., the jumping movements. The only far‐reaching impact on the organisation of these fibres had the formation of a circular adoral zone. Its introduction seems to co‐occur with that of two microtubular rings (adoral fibre and preoral ring) at the expense of the tuft‐shaped submembranellar fibre. The nematodesmata originating in the polykinetids apparently generate various, possibly phylogenetically significant patterns. This feature complex and the discovery of several characters in the present study necessitate further ultrastructural investigations in all taxa discussed here.

## Supporting information


**Figure S1.** *Schmidingerella meunieri*, transmission electron micrographs of longitudinal sections of the anterior cell portion showing the successive opening of the buccal cavity.
**Figure S2.** *Schmidingerella meunieri*, transmission electron micrographs of cross and oblique sections of collar membranelles and associated structures.
**Figure S3.** *Schmidingerella meunieri*, transmission electron micrographs of cross and oblique longitudinal sections of the collar membranelles’ inner and outer portions.
**Figure S4.** *Schmidingerella meunieri*, transmission electron micrographs of cross and longitudinal sections of collar polykinetids.
**Figure S5.** *Schmidingerella meunieri*, transmission electron micrographs of slightly oblique cross sections of a single collar membranelle at different levels.
**Figure S6.** *Schmidingerella meunieri*, transmission electron micrographs of longitudinal and oblique sections of collar membranelles showing the lateral microtubules.
**Figure S7.** *Schmidingerella meunieri*, transmission electron micrographs of longitudinal sections of the anterior cell portion showing the adoral fibre and preoral ring.
**Figure S8.** *Schmidingerella meunieri*, transmission electron micrographs of longitudinal and cross sections of the outer and inner portions of collar membranelles.
**Figure S9.** *Schmidingerella meunieri*, transmission electron micrographs of oblique longitudinal sections showing the middle portions of collar polykinetids and their associated nematodesmata.
**Figure S10.** *Schmidingerella meunieri*, transmission electron micrographs of longitudinal sections of the cell proper.
**Figure S11.** *Schmidingerella meunieri*, transmission electron micrographs of longitudinal sections.
**Figure S12.** *Schmidingerella meunieri*, transmission electron micrographs of cross‐sections of the stichomonad endoral membrane.
**Figure S13.** *Schmidingerella meunieri*, transmission electron micrographs of longitudinal sections of the anterior cell portion showing the distal portion of the endoral membrane.
**Figure S14.** *Schmidingerella meunieri*, transmission electron micrographs of longitudinal and cross sections of microtubular bundles underpinning the peristomial field.
**Figure S15.** Anterior cell portion of the congener *Schmidingerella arcuata* after protargol staining.
**Figure S16.** *Schmidingerella meunieri*, transmission electron micrographs of longitudinal and cross‐sections focussing on structures associated with the buccal cavity.
**Figure S17.** *Schmidingerella meunieri*, video of rotating 3D reconstruction (made with software SketchUp Pro) of an adoral polykinetid comprising three rows and five files of basal bodies.
**Figure S18.** *Schmidingerella meunieri*, video of rotating 3D reconstruction (made with software Blender) of the anterior cell portion showing the adoral zone of membranelles and associated microtubular bundles.
