# Supplementary material for: Ultrastructural Studies on a Model Tintinnid – Schmidingerella meunieri (Kofoid & Campbell, 1929) Agatha & Strüder‐Kypke, 2012 (Ciliophora). II. The Oral Apparatus
Source: J Eukaryot Microbiol. 2020 May 11;67(4):463–79. doi: 10.1111/jeu.12795 (PMC7384128; doi:10.1111/jeu.12795)
Supplement: Supplementary file 1 — Figure S1. Schmidingerella meunieri, transmission electron micrographs of longitudinal sections of the anterior cell portion showing the successive opening of the buccal cavity. Figure S2. Schmidingerella meunieri, transmission electron micrographs of cross and oblique sections of collar membranelles and associated structures. Figure S3. Schmidingerella meunieri, transmission electron micrographs of cross and oblique longitudinal sections of the collar membranelles’ inner and outer portions. Figure S4. Schmidingerella meunieri, transmission electron micrographs of cross and longitudinal sections of collar polykinetids. Figure S5. Schmidingerella meunieri, transmission electron micrographs of slightly oblique cross sections of a single collar membranelle at different levels. Figure S6. Schmidingerella meunieri, transmission electron micrographs of longitudinal and oblique sections of collar membranelles showing the lateral microtubules. Figure S7. Schmidingerella meunieri, transmission electron micrographs of longitudinal sections of the anterior cell portion showing the adoral fibre and preoral ring. Figure S8. Schmidingerella meunieri, transmission electron micrographs of longitudinal and cross sections of the outer and inner portions of collar membranelles. Figure S9. Schmidingerella meunieri, transmission electron micrographs of oblique longitudinal sections showing the middle portions of collar polykinetids and their associated nematodesmata. Figure S10. Schmidingerella meunieri, transmission electron micrographs of longitudinal sections of the cell proper. Figure S11. Schmidingerella meunieri, transmission electron micrographs of longitudinal sections. Figure S12. Schmidingerella meunieri, transmission electron micrographs of cross‐sections of the stichomonad endoral membrane. Figure S13. Schmidingerella meunieri, transmission electron micrographs of longitudinal sections of the anterior cell portion showing the distal portion of the endoral membrane. Figure S1 [file JEU-67-463-s001.pdf]

SUPPORTING INFORMATION

Ultrastructural Studies on a Model Tintinnid - *Schmidingerella meunieri* (Kofoid & Campbell, 1929) Agatha & Strüder-Kypke, 2012 (Ciliophora). II. The Oral Apparatus by Michael S. Gruber, Birgit Weissenbacher, Sabine Agatha

Figure S1 *Schmidingerella meunieri*, transmission electron micrographs of longitudinal sections of the anterior cell portion showing the successive opening of the buccal cavity. (**A**) Section dorsal of the buccal opening. Note the symmetry of the structures in the peristomial rim. (**B**) Proximal portion of the obliquely orientated, funnel-shaped buccal cavity. The proximal cilia of the endoral membrane covered by membranous layers (possibly perilemma) and the cilia of the proximalmost membranelles extend deep into the buccal cavity. Polygonal microtubular bundles (arrows) are arranged around the cavity. (**C**) Section close to the overture of the buccal cavity showing the proximal portion of the endoral membrane with its curved cilia. Cross sections of the polygonal bundles (arrows) are still visible. (**D**) Left cell portion showing the slope of the peristomial field. Two membranelles (possibly the buccal and one elongated collar membranelle) separated by a ridge extend on the longitudinal ventral wall of the buccal cavity. AF, adoral fibre; BM, buccal membranelle; CM, collar membranelles; ECM, elongated collar membranelles; EM, endoral membrane; IR, intermembranellar ridges; LMt, lateral microtubules; Ma, macronuclear nodules; ML, membranous layers probably also enclosing cytoplasm; NeP, nematodesmata originating in outer portions of collar polykinetids; P, perilemma; PR, preoral ring. Scale bars = 5 µm (A, C), 3 µm (B), 10 µm (D).

Figure S2 *Schmidingerella meunieri*, transmission electron micrographs of cross and oblique sections of collar membranelles and associated structures. (**A**) A stria containing extrusomes extends longitudinally along a collar membranelle. The perilemma (arrows) encloses both stria and membranelle. (**B**) An intermembranellar ridge containing numerous granules (possibly extrusive organelles) and several vacuoles with fluffy content. Lateral microtubules extend through the intermembranellar ridge to the adjacent collar membranelle. (**C**) A tentaculoid inserting on an intermembranellar ridge. It contains two kinds of probably extrusive organelles, i.e., capsules and mucocysts. CM, collar membranelles; Ex, extrusomes; IR, intermembranellar ridges; LMt, lateral microtubules; M, mitochondria; S, striae; T, tentaculoids. Scale bars = 1 µm (A, C), 2 µm (B).

Figure S3 *Schmidingerella meunieri*, transmission electron micrographs of cross and oblique longitudinal sections of the collar membranelles' inner and outer portions. (**A**) Cross section displaying one condylocilium at the inner end of row 3 and three condylocilia in row 2; row 1 has no condylocilia. (**B**) Oblique longitudinal section of an outer portion showing at least two condylocilia per row. Note the aligned lateral microtubules (arrow) originating from basal bodies in another sectional plane. (**C**) Cross section of the stepped inner end. (**D**) Cross section of proximal basal body portions. Only the cytoplasm between the rows contains vacuoles (arrows). 1-3, rows 1-3 of basal bodies or cilia; ALC, anterior longitudinal connections; Ax, axosomes; CG, core granules; CM, collar membranelles; Co, condylocilia; Cw, cartwheels; DLC, diagonal longitudinal connections; P, perilemma; PLC, posterior longitudinal connections; SPC, single posterior connection. Scale bars = 1 µm (A, B), 2 µm (C), 500 nm (D).

Figure S4 *Schmidingerella meunieri*, transmission electron micrographs of cross and longitudinal sections of collar polykinetids. (**A**) Cross section showing fifteen basal bodies sectioned at different levels. Electron-dense connections link the A-tubules (arrow) as well as the A- and C-tubules (arrowhead) of adjacent microtubular triplets. (**B**) Longitudinal section of eight basal bodies showing the proximal cartwheel, the electron-dense core granules, and the axosome. 1-3, basal body rows 1-3; Ax, axosomes; CG, core granules; Cw, cartwheels; DC, diagonal connections; DM, electron-dense matters; OC1, oblique connections 1. Scale bars = 500 nm.

Figure S5 *Schmidingerella meunieri*, transmission electron micrographs of slightly oblique cross sections of a single collar membranelle at different levels. (**A, B**) Sections mainly on cartwheel level. Electron-dense material connects the basal bodies in each row. Vacuoles are found exclusively between the rows. Lateral microtubules originate in bulges of electron-dense matter at the posterior margin of the polykinetid (cp. Fig. 2, S6A, B, D) and are arranged in fan-shaped patterns perpendicular to the basal bodies' main axes (B). Apparently, they touch the electron-dense hooks at the anterior side of the following polykinetid (arrow; B). (**C**, **D**) Sections of the inner polykinetid portion at the level of the core granules and axosomes. Condylocilia are restricted to the innermost basal bodies of rows 2 and 3. The basal bodies of row 1 are tangentially linked by the single posterior connection. Most connections between the basal bodies are indistinct due to their oblique courses. The vacuoles between the rows almost disappeared at this level. 1-3, basal body rows 1-3; AC, anterior connections; ALC, anterior longitudinal connections; Ax, axosomes; CG, core granules; Co, condylocilia; Cw, cartwheels; DC, diagonal connections; DLC, diagonal longitudinal connections; DM, electron-dense matter; H, hook-shaped structures; LMt, lateral microtubules; LTC, left transverse connections; OC1, oblique connections 1; OC2, oblique connections 2; PLC, posterior longitudinal connections; PMC, postmembranellar connections; SPC, single posterior connection. Scale bars = 500 nm.

Figure S6 *Schmidingerella meunieri*, transmission electron micrographs of longitudinal (**A, C**) and oblique (**B, D**) sections of collar membranelles showing the lateral microtubules. Equidistantly spaced lateral microtubules originate from electron-dense matter along the basal bodies of row 1. They extend to electron-dense, hook-shaped structures at the basal bodies in row 3 of the following polykinetid. 1-3, rows 1-3 of basal bodies or cilia; Ax, axosomes; CG, core granules; CT, central microtubules; Cw, cartwheels; DC, diagonal connections; DM, electron-dense matter; H, hook-shaped structures; LMt, lateral microtubules; P, perilemma. Scale bars = 500 nm (A, C, D), 1 µm (B).

Figure S7 *Schmidingerella meunieri*, transmission electron micrographs of longitudinal sections of the anterior cell portion showing the adoral fibre and preoral ring. (**A**) Nematodesmata originating in the outer polykinetid portions (right polykinetid) form thick bundles, which extend obliquely posteriorly in clockwise and anti-clockwise directions and merge with the adoral fibre. Those of the polykinetids' middle portions (middle polykinetid) extend over a short distance obliquely posteriorly and merge with the preoral ring. (**B**) Longitudinal and cross sections of several microtubules (arrow) indicate that nematodesmata from the middle portions of adjacent polykinetids meet at the preoral ring. Note the hook-shaped structure at the basal body of row 3 and the lateral microtubules originating from the posterior side (row 1) of the polykinetid. 1, row 1 of basal bodies or cilia; AF, adoral fibre; CM, collar membranelles; H, hook-shaped structures;

LMt, lateral microtubules; M, mitochondria; NeP, nematodesmata of adoral polykinetids; PR, preoral ring. Scale bars = 5 µm (A), 2 µm (B).

Figure S8 *Schmidingerella meunieri*, transmission electron micrographs of longitudinal and cross sections of the outer and inner portions of collar membranelles. (**A**) Detail of the membranelles' outer portion. The nematodesmata of at least two basal body rows extend in two main directions, causing the conspicuous zigzagging pattern easily recognisable in protargol-stained specimens (cp. 3, S15B). (**B**) The nematodesmata originating in the outer portion of row 3 form long bundles, which extend obliquely posteriorly, before merging with the adoral fibre. Note the condylocilium in row 1. (**C**) Nematodesmata originating from the inner portion extend posteriorly and merge with the adoral fibre (seen from inside the peristomial rim). (**D**) Inner portion of a collar membranelle seen from the peristomial field. In this case, the nematodesmata from row 1 merge with the adoral fibre whose microtubules are sectioned in different angles. 1-3, rows 1-3 of basal bodies or cilia; AF, adoral fibre; Co, condylocilia; DM, electron-dense matter; H, hook-shaped structures; IR, intermembranellar ridges; LMt, lateral microtubules; NeP, nematodesmata of adoral polykinetids; PR, preoral ring; SPC, single posterior connection; T, tentaculoids. Scale bars = 1 µm (A, C, D), 5 µm (B).

Figure S9 *Schmidingerella meunieri*, transmission electron micrographs of oblique longitudinal sections showing the middle portions of collar polykinetids and their associated nematodesmata. (**A**) All basal bodies of a file have associated nematodesmata, which extend in angles of up to 90° to each other posteriorly, before merging with the preoral fibre. (**B**) A basal body row. 1-3, rows 1-3 of basal bodies or cilia; LMt, lateral microtubules; NeP, nematodesmata of adoral polykinetids; PR, preoral ring. Scale bars = 1 µm (A), 2 µm (B).

Figure S10 *Schmidingerella meunieri*, transmission electron micrographs of longitudinal sections of the cell proper. (**A**) Microtubular bundles (arrows) originating in the adoral fibre and the distal portion of the endoral membrane (cp. Fig. S12E, S13A, B, S15A) fuse, forming the dorsal fibre bundle, which extends to the myoneme in the posterior cell portion. (**B**) Detail of the meeting point of the dorsal fibre bundle and the myoneme. CM, dorsal collar membranelles; DFB, dorsal fibre bundle; Ma, macronuclear nodules; My, myoneme; NeP, nematodesmata of adoral polykinetids. Scale bars = 10 µm (A), 5 µm (B).

Figure S11 *Schmidingerella meunieri*, transmission electron micrographs of longitudinal sections. (**A**) Posterior portion of cell proper. The ventral fibre bundles (arrows) are formed by nematodesmata originating in the inner portions of the buccal polykinetid and probably three elongated collar polykinetids. They probably remain separate and terminate - like the dorsal bundle - at the myoneme (not shown). (**B**) Inner portions of two elongated collar membranelles seen from the ventral wall of the buccal cavity. Their nematodesmata form the ventral fibre bundles (arrow). 1-3, rows 1-3 of basal bodies or cilia; BC, buccal cavity; ECM, elongated collar membranelles. Scale bars = 5 µm (A), 3 µm (B).

Figure S12 *Schmidingerella meunieri*, transmission electron micrographs of cross sections of the stichomonad endoral membrane. (**A, B**) Oblique sections of basal bodies in the region between axosome and cartwheel (A) and of the proximal portions of the cilia (B). Bundles of three or four microtubules extend parallel to the left sides of the basal bodies and the proximal cilia portions

(arrows). Microtubules probably originating from the adoral fibre extend obliquely posteriorly to merge with the dorsal fibre bundle. (**C**) The cilia extend across the peristomial field. They are enclosed with perilemma and covered by additional membranous layers probably also enclosing cytoplasm. The microtubular triplets and quadruplets originating on the left sides of the basal bodies are short and thus already terminated. (**D**) Thin strips (arrowheads) of electron-dense material extend on both sides of the basal body row. Microtubular bundles originate in electron-dense material (arrows) on both sides of the basal bodies. (**E**) Distal portion of the endoral membrane. Microtubules originating from the right sides of the basal bodies extend anteriorly, merging with the adoral fibre. Those from the left sides form triplets or quadruplets underpinning the peristomial field (arrows). Apparently, additional bundles extend posteriorly, forming the dorsal fibre bundle together with microtubules originating in the adoral fibre (arrowhead). AP, axosomal plates; Ax, axosomes; CG, core granules; Ci, cilia; Cw, cartwheels; EMt, endoral microtubular bundles; ML, membranous layers probably also enclosing cytoplasm; Mt, microtubules probably originating in the adoral fibre; NeE?, perhaps nematodesmata of endoral membrane; P, perilemma; V, vacuoles. Scale bars = 500 nm (A, C-E), 200 nm (B).

Figure S13 *Schmidingerella meunieri*, transmission electron micrographs of longitudinal sections of the anterior cell portion showing the distal portion of the endoral membrane (**A, B**). The microtubules commencing at the right sides of the basal bodies are distinctly longer than those in the remaining endoral due to the enlarged distance to the adoral fibre. On the left side of the endoral basal bodies not only the microtubular triplets and quadruplets underpinning the peristomial field commence but also bundles extending posteriorly and joining the microtubules originating in the adoral fibre (arrow) to form the dorsal fibre bundle. The cracks in the dorsal fibre bundle are artifacts. 1, ciliary row 1; AF, adoral fibre; CM, collar membranelles; Con, concavity formed by peristomial rim; DFB, dorsal fibre bundle; EM, endoral membrane; EMt, endoral microtubular bundles; LMt, lateral microtubules; NeE?, perhaps nematodesmata of endoral membrane; NeP, nematodesmata of adoral polykinetids; SC, somatic cilia. Scale bars = 5 µm (A), 10 µm (B).

Figure S14 *Schmidingerella meunieri*, transmission electron micrographs of longitudinal (A, B) and cross sections (C, D) of microtubular bundles underpinning the peristomial field. (**A**, **B**) The microtubules originate in the adoral fibre (A) and soon form polygonal bundles (B) extending horizontally towards the buccal cavity. The membranous layers covering the endoral membrane enclose material of unknown nature (asterisk; probably an artifact). (**C**) Equally spaced arrangement of the polygonal bundles near the buccal cavity's overture. Finally, the polygonal bundles encompass the buccal cavity on three sides (cp. Fig. S1B, C). (**D**) Cross sections of the polygonal bundles at higher magnification showing up to 58 highly ordered microtubules each. 1-3, rows 1-3 of basal bodies or cilia; AF, adoral fibre; Ci, cilia; CM, collar membranelles; ECM, elongated collar membranelles; EM, endoral membrane; IR, intermembranellar ridges; Mt, microtubules originating in adoral fibre; P, perilemma; PB, polygonal bundles; T, tentaculoids. Scale bars = 2 µm (A-C), 500 nm (D).

Figure S15 Anterior cell portion of the congener *Schmidingerella arcuata* after protargol staining. (**A**) Detail showing the microtubular bundles (asterisks) extending (i) between the adoral fibre and the endoral membrane, (ii) from the endoral membrane posteriorly, and (iii) from the adoral fibre posteriorly, forming the dorsal fibre bundle. A further bundle (arrowhead)

originates in the distal portion of the endoral membrane and curves in clockwise direction close underneath the dorsal cell surface into the peristomial rim; possibly, it consists of endoral nematodesmata. (**B**) Nematodesmata originating in the collar membranelles' outer portions form a prominent zigzagging pattern before they merge with the adoral fibre. AF, adoral fibre; CM, collar membranelles; DFB, dorsal fibre bundle; EM, endoral membrane; Ma, macronuclear nodules; NeP, nematodesmata of adoral polykinetids; SC, somatic ciliature. Scale bars = 5 µm.

Figure S16 *Schmidingerella meunieri*, transmission electron micrographs of longitudinal and cross sections focussing on structures associated with the buccal cavity. (**A**) Longitudinal cell section. The endoral membrane is distinctly apart from the peristomial field (arrow) suggesting that the conspicuous pumping movements of the peristomial area visible in live specimens are caused by its beating cilia. Note the inclination of the peristomial field towards the buccal cavity. (**B**) Detail of Figure S1A showing the inner portion of a collar membranelle. The nematodesmata originate from its basal body row 2 (arrow). (**C**) The microtubules constituting the adoral fibre are linked by electron-dense threads (arrow). (**D**) Assemblage of microtubules of unknown origin right at the entrance to the buccal cavity (possibly pharyngeal fibres; arrow). (**E**) Microtubules with unknown origin extending in an intermembranellar ridge at the entrance to the buccal cavity. 1, ciliary row 1; AF, adoral fibre; BC, buccal cavity; CM, collar membranelles; ECM, elongated collar membranelles; EM, endoral membrane; LMt, lateral microtubules; Mt, microtubules; NeP, nematodesmata of adoral polykinetids; PB, polygonal bundles; PF, peristomial field. Scale bars = 15 µm (A), 2 µm (B), 100 nm (C), 1 µm (D, E).

Figure S17 *Schmidingerella meunieri*, video of rotating 3D reconstruction (made with software SketchUp Pro) of an adoral polykinetid comprising three rows and five files of basal bodies (cp. Fig. 1A). The dark grey proximal portions of the basal bodies represents the cartwheel regions, the light grey portions comprise the core granule regions and the axosomes (Ax). The internal connections are labelled by colours: orange = left transverse connections (LTC); light green = single posterior connection (SPC) with paired bulges of electron-dense matter (DM) extending along the basal bodies; purple = oblique connections 1 (OC1); dark blue = oblique connections 2 (OC2); brown = hook-shaped structures (H); dark green = diagonal connections (DC); yellow = anterior connections (AC); yellowish brown = postmembranellar connections (PMC); colourless pattern at proximal end of basal bodies = posterior, diagonal, and anterior longitudinal connections (ALC, DLC, PLC); white rods = lateral microtubules (LMt) at posterior polykinetid margin.

Figure S18 *Schmidingerella meunieri*, video of rotating 3D reconstruction (made with software Blender) of the anterior cell portion showing the adoral zone of membranelles and associated microtubular bundles. Note that the lateral microtubules linking adjacent polykinetids are not shown for the sake of clarity. The oral structures are labelled by colours: yellow = three-rowed polykinetids; dark blue = adoral fibre; light blue = nematodesmata originating from the outer and inner portions of the collar polykinetids; dark orange = preoral ring; light orange = nematodesmata originating from the middle portions of the collar polykinetids; dark grey = ventral and dorsal fibre bundles; light grey = peristomial rim, peristomial field, and buccal cavity.

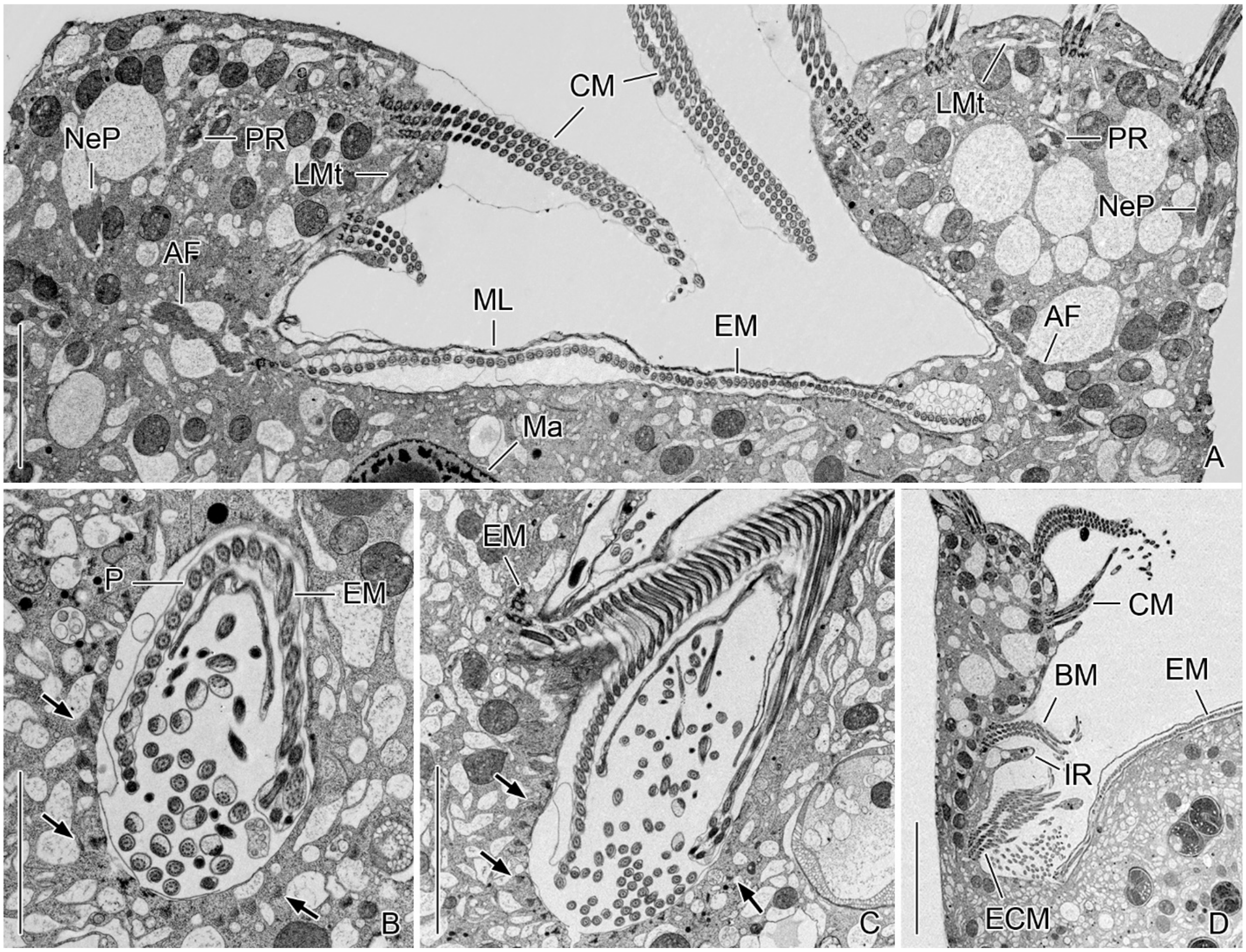
CM
NeP
PR
LMt
AF
ML
EM
Ma
LMt
PR
NeP
AF
A
P
EM
B
EM
C
CM
BM
EM
IR
ECM
D

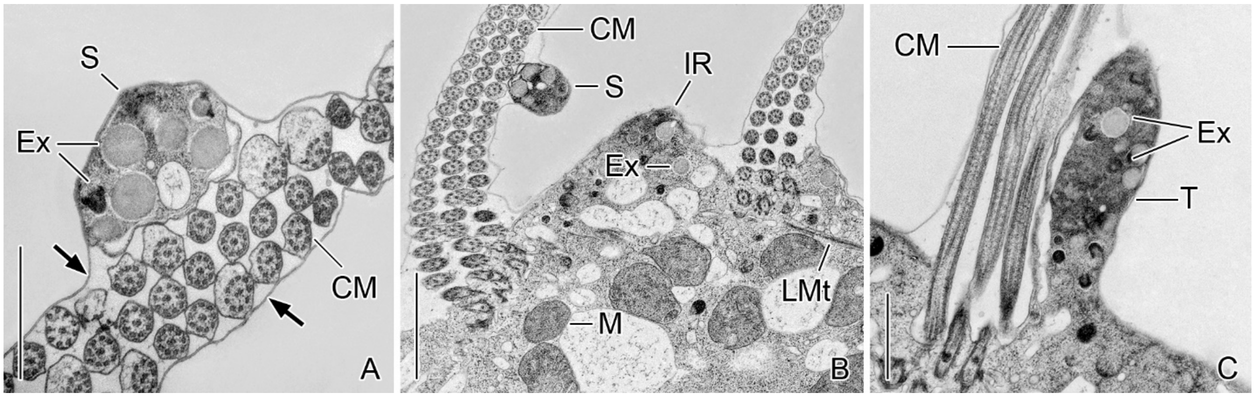
S
Ex
CM
A
CM
S
IR
Ex
LMt
M
B
CM
Ex
T
C

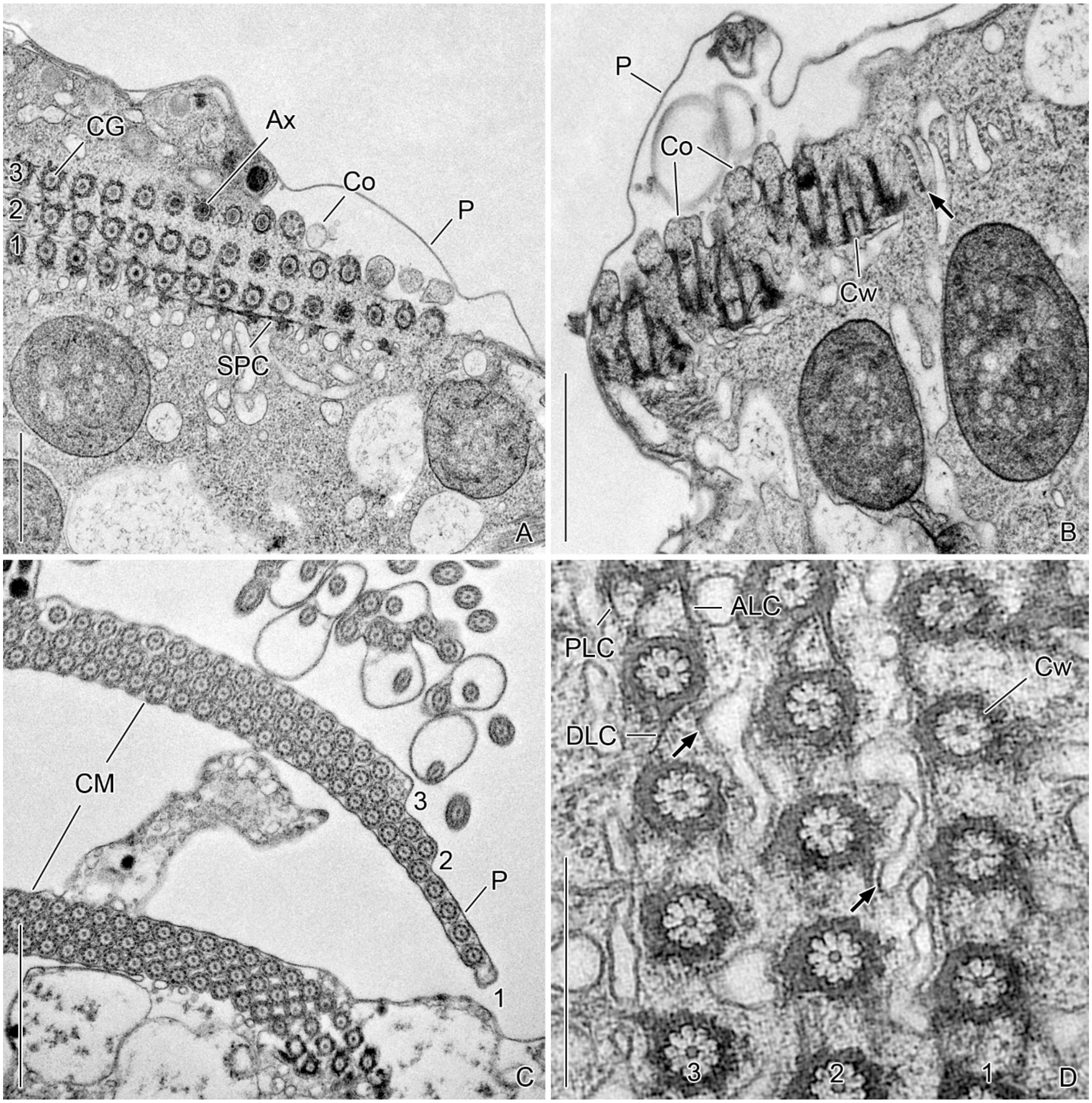
CG
Ax
Co
P
3
2
1
SPC
A
P
Co
Cw
B
CM
CM
3
2
P
1
C
ALC
PLC
Cw
DLC
3
2
1
D

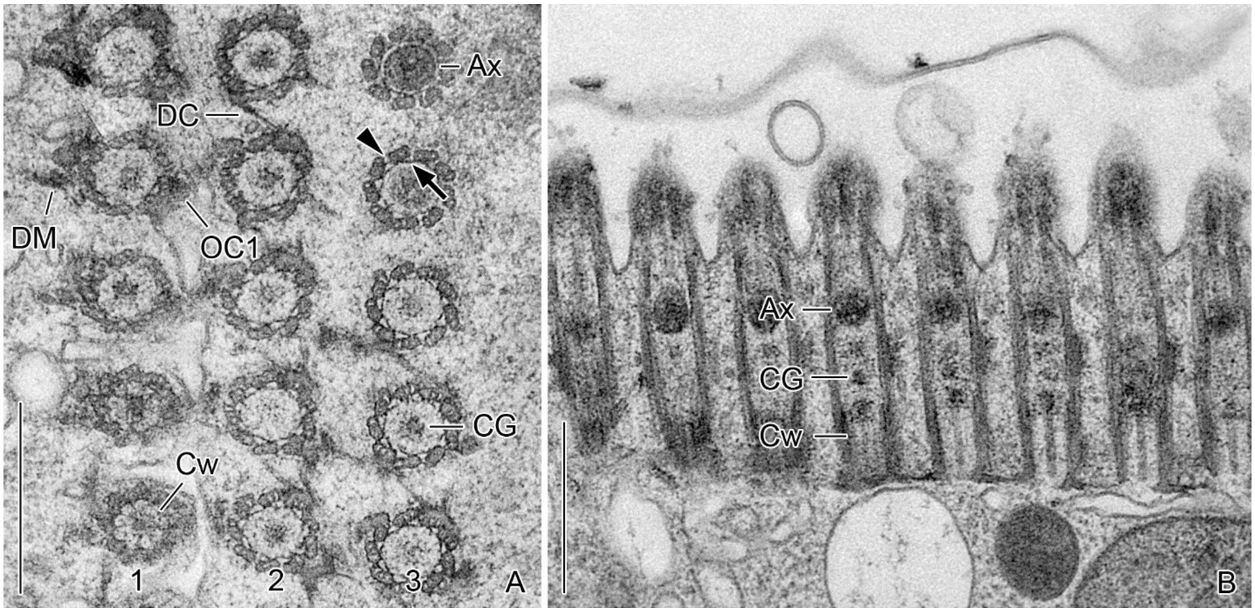
Ax
DC
DM
OC1
CG
Cw
1
2
3
A
Ax
CG
Cw
B

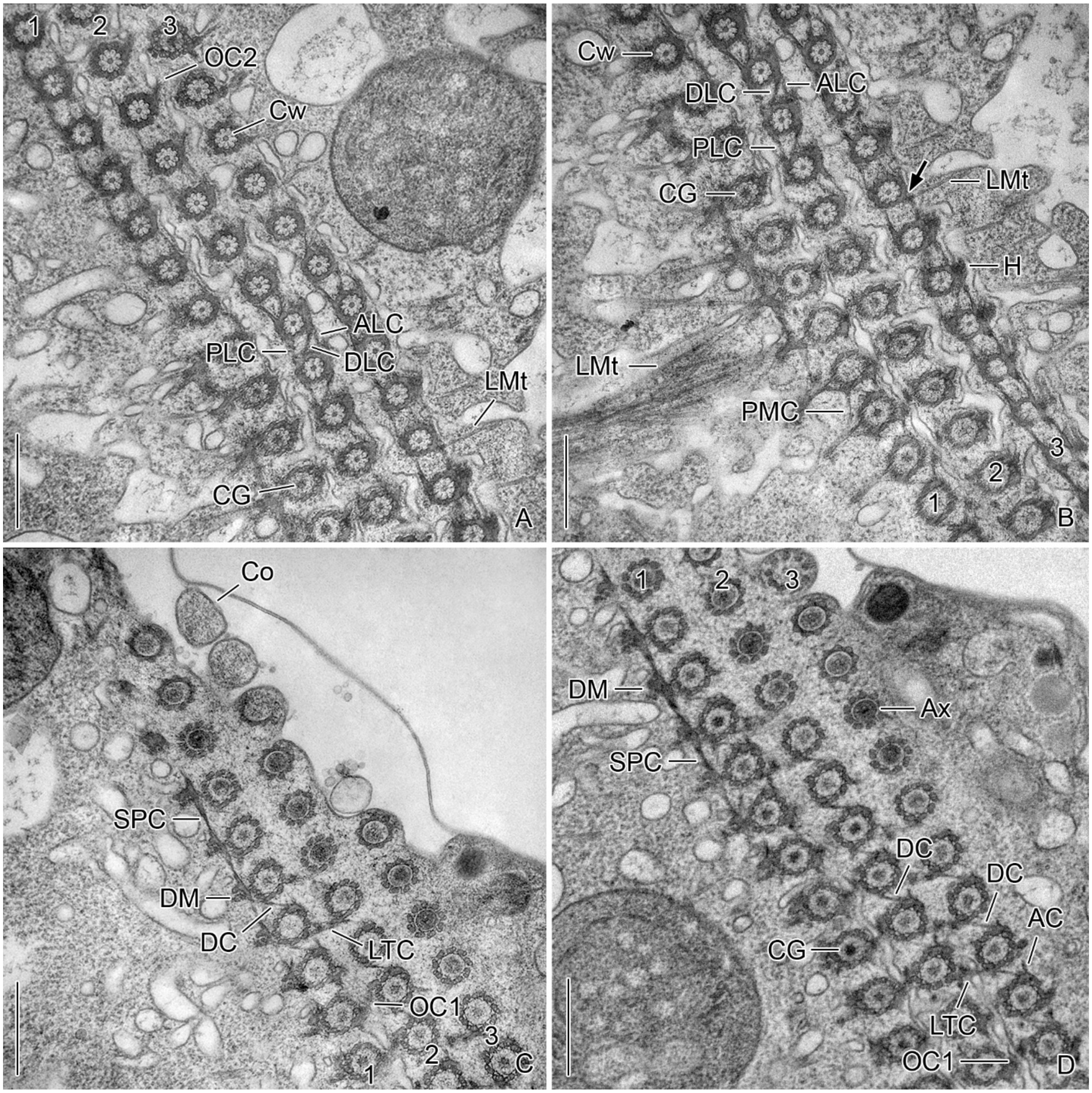
1
2
3
OC2
Cw
ALC
PLC
DLC
LMt
CG
A
Cw
DLC
ALC
PLC
LMt
CG
H
LMt
PMC
3
2
1
B
Co
SPC
DM
DC
LTC
OC1
3
2
1
C
1
2
3
DM
Ax
SPC
DC
DC
AC
CG
LTC
OC1
D

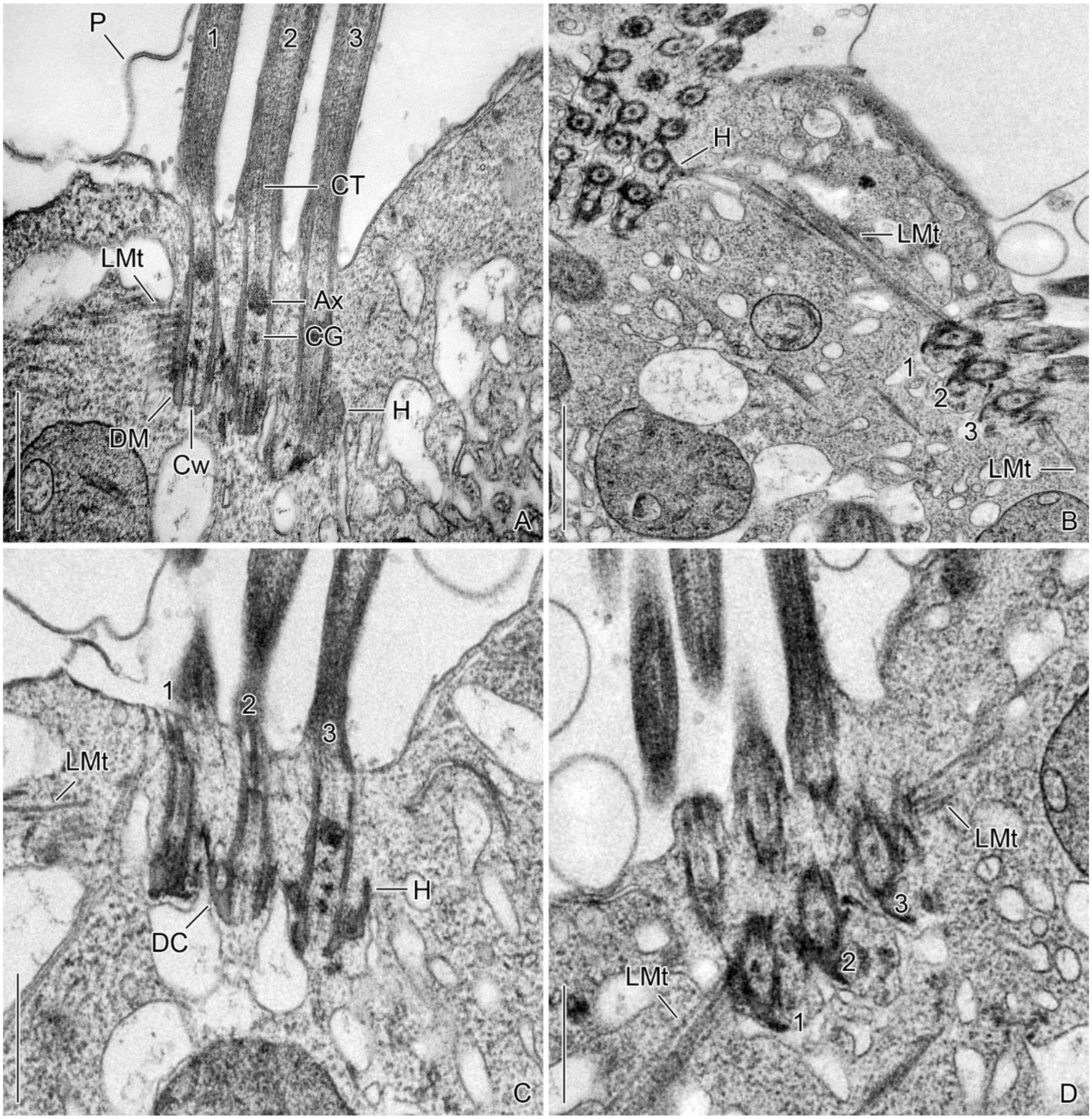
P
1
2
3
CT
LMt
Ax
CG
H
DM
Cw
A
H
LMt
1
2
3
LMt
B
1
2
3
LMt
H
DC
C
LMt
3
2
LMt
1
D

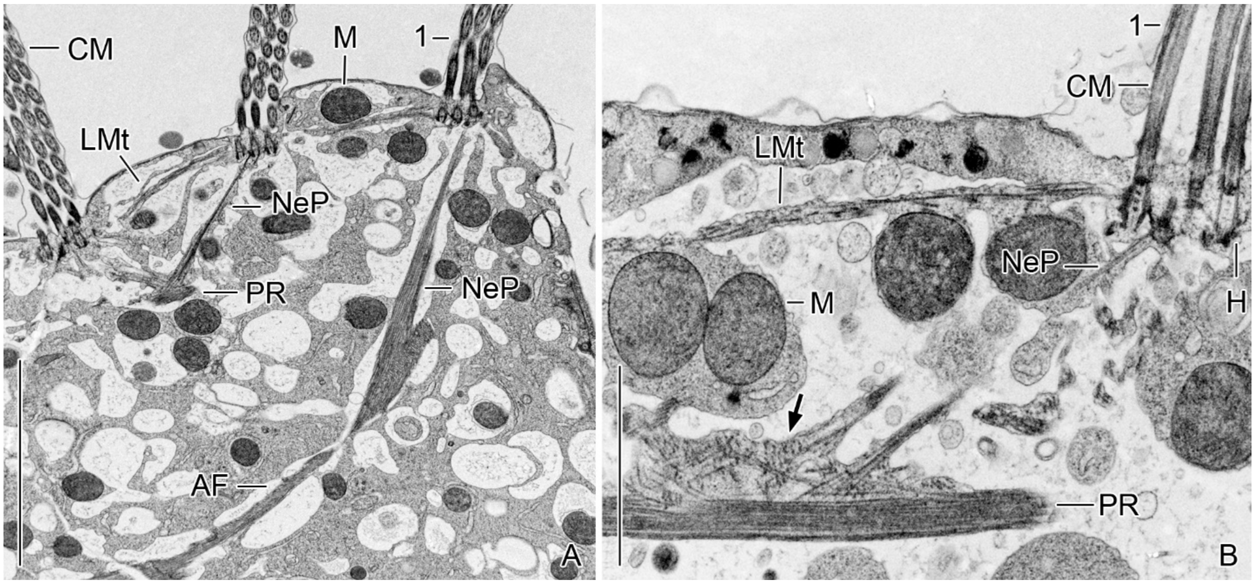
CM
M
1
LMt
NeP
PR
NeP
AF
A
1
CM
LMt
NeP
H
M
PR
B

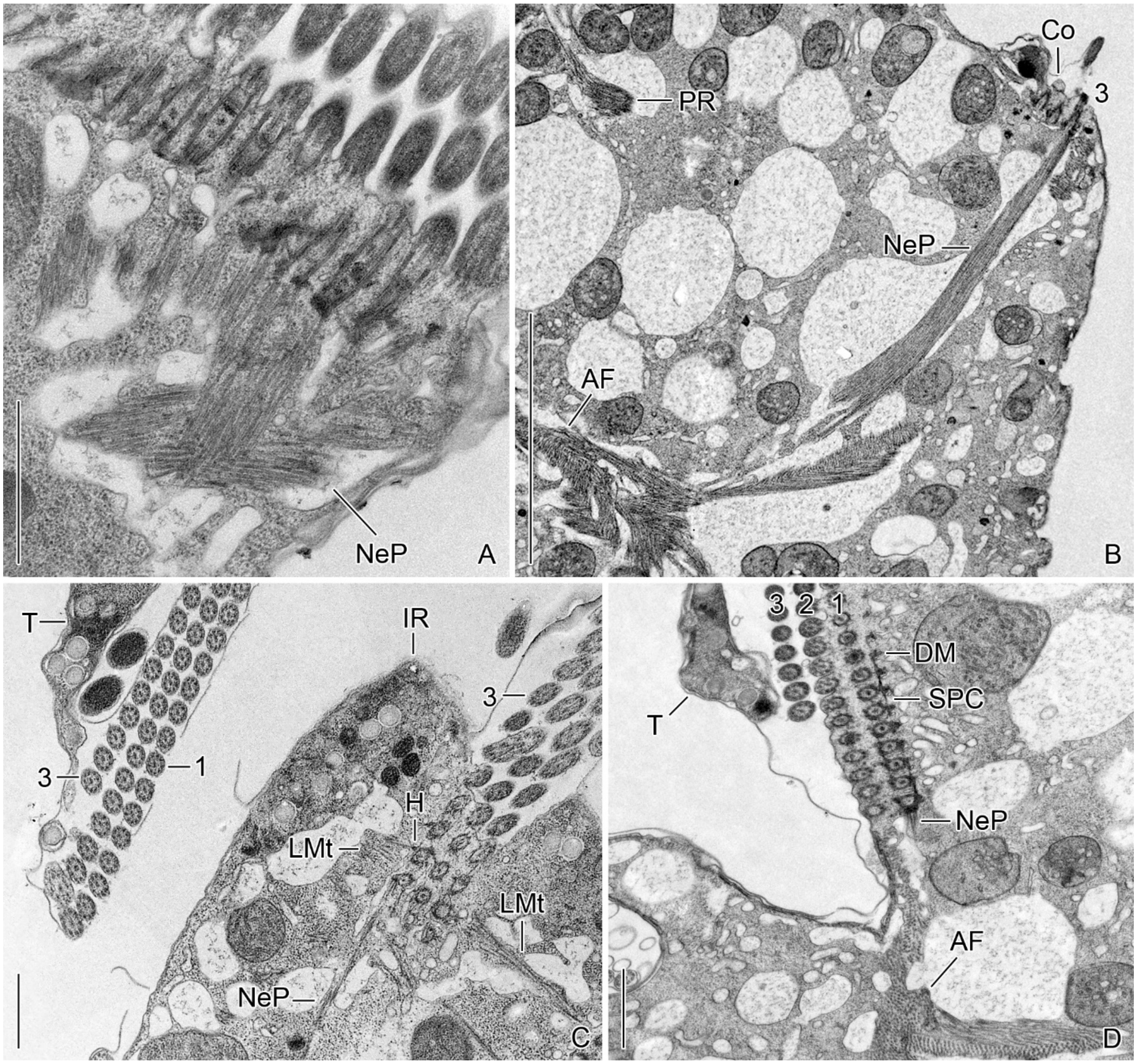
NeP
A
Co
PR
3
NeP
AF
B
T
IR
3
1
3
H
LMt
LMt
NeP
C
3 2 1
DM
T
SPC
NeP
AF
D

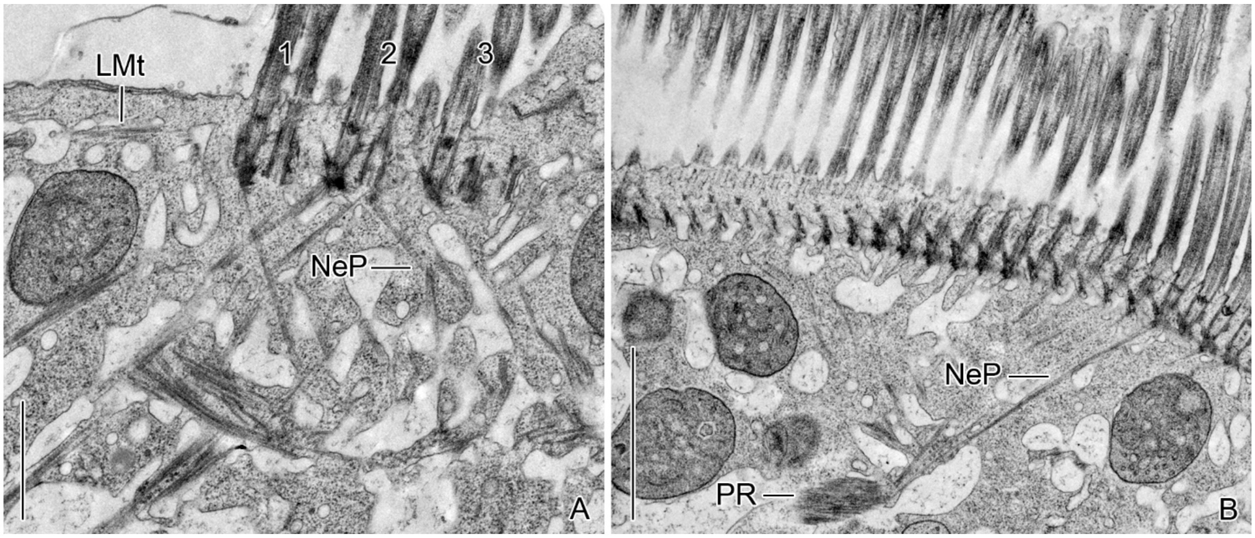
LMt
1
2
3
NeP
A
NeP
PR
B

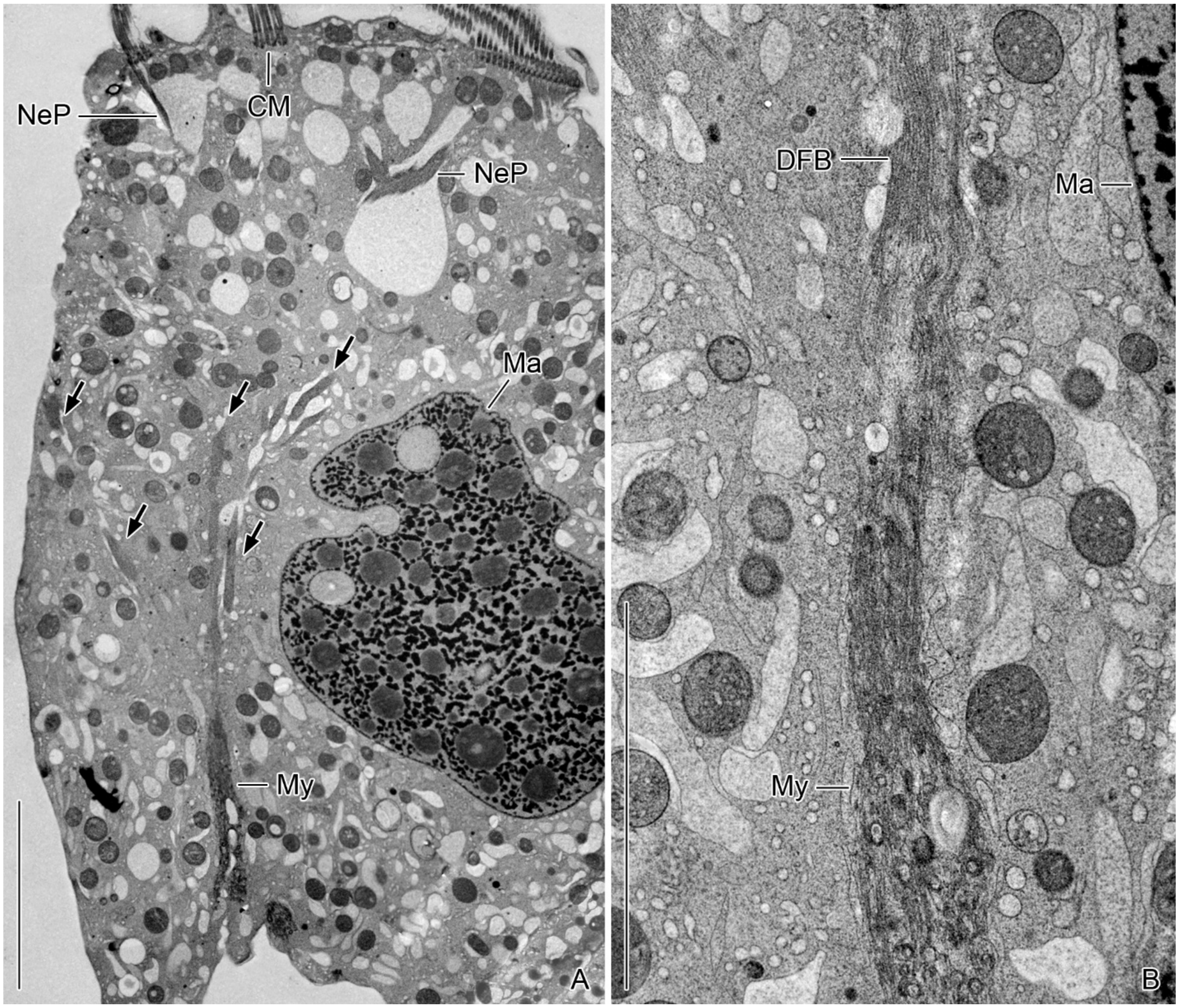
NeP
CM
NeP
Ma
My
A
DFB
Ma
My
B

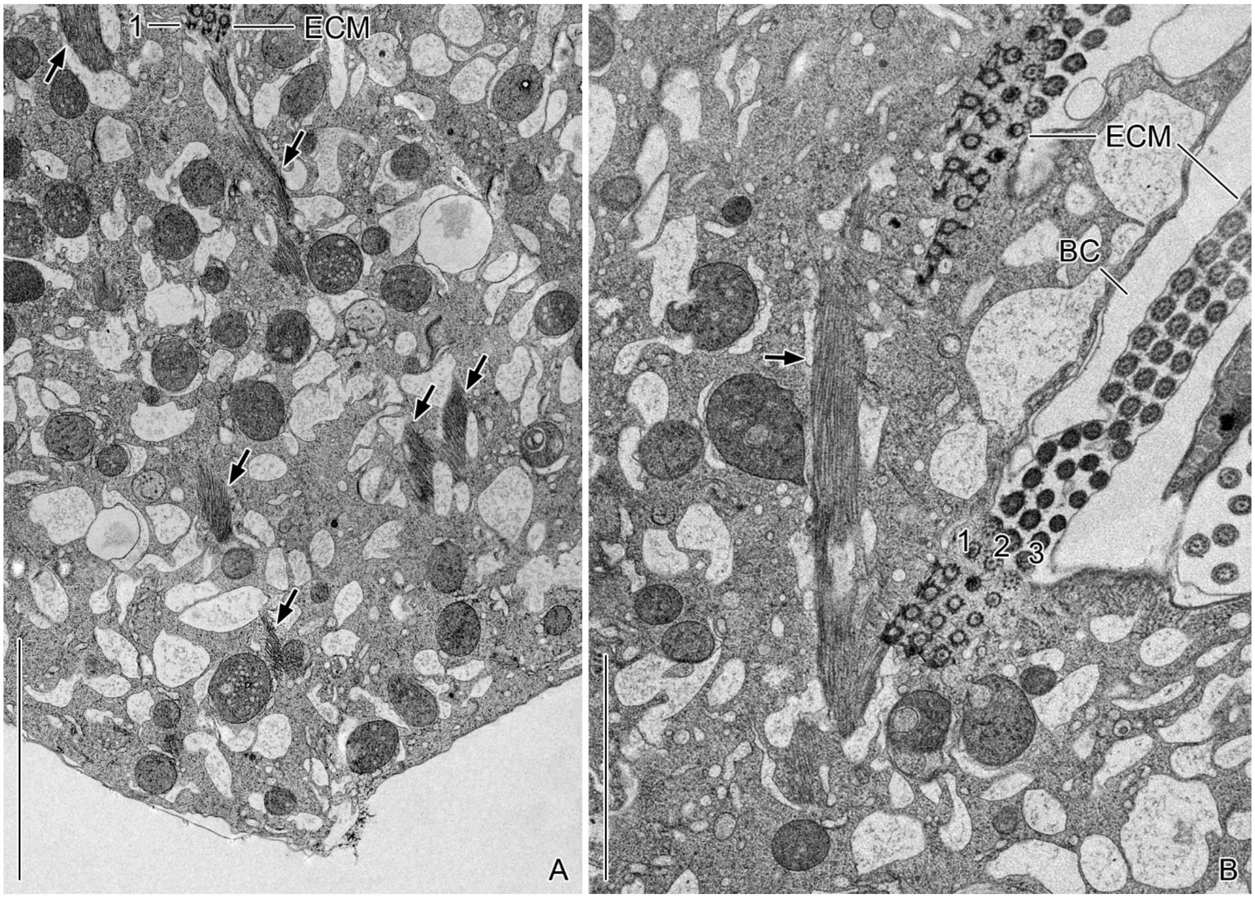
1
ECM
A
ECM
BC
1
2
3
B

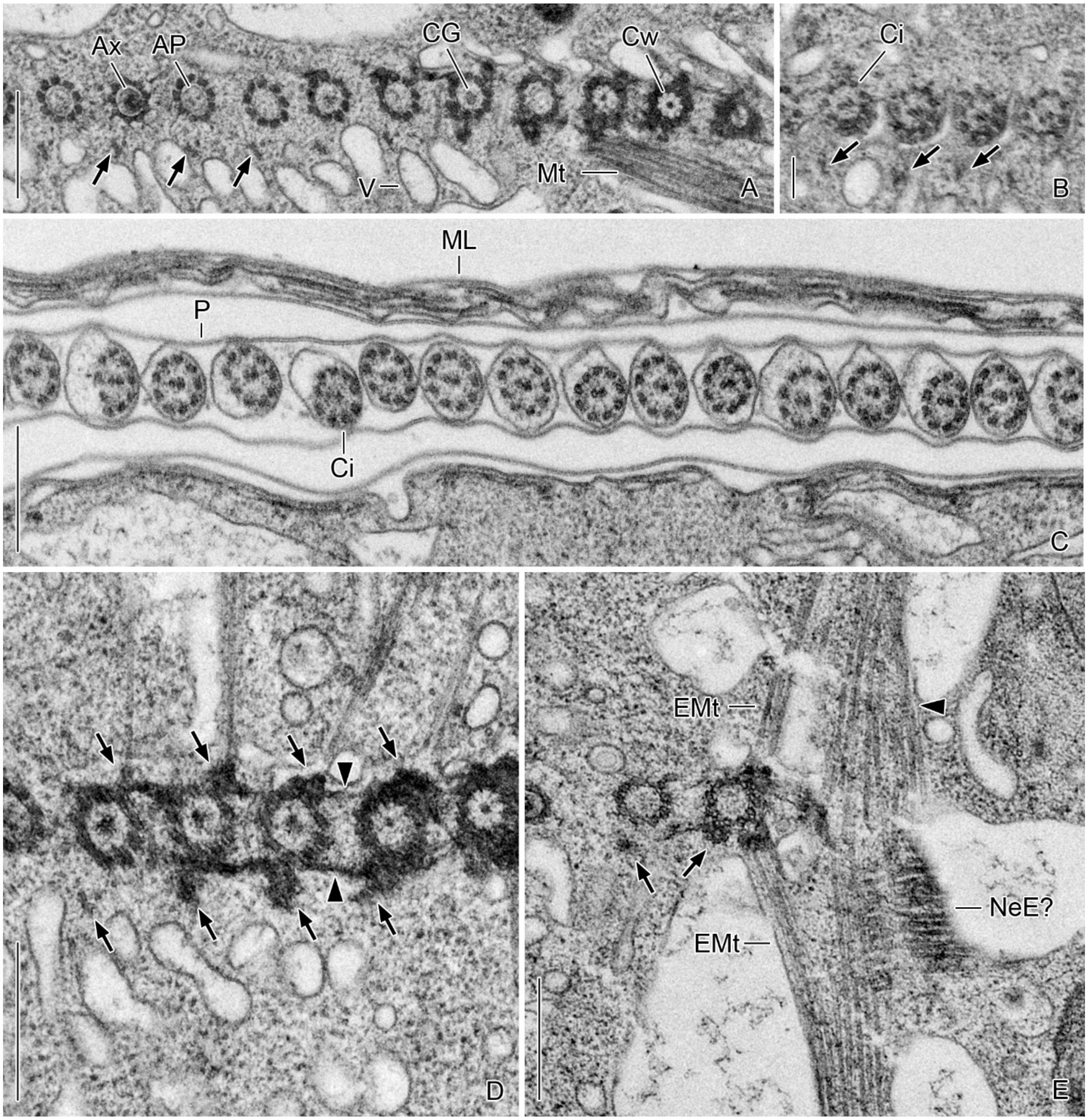
Ax
AP
CG
Cw
V
Mt
A
Ci
B
ML
P
Ci
C
D
EMt
EMt
NeE?
E

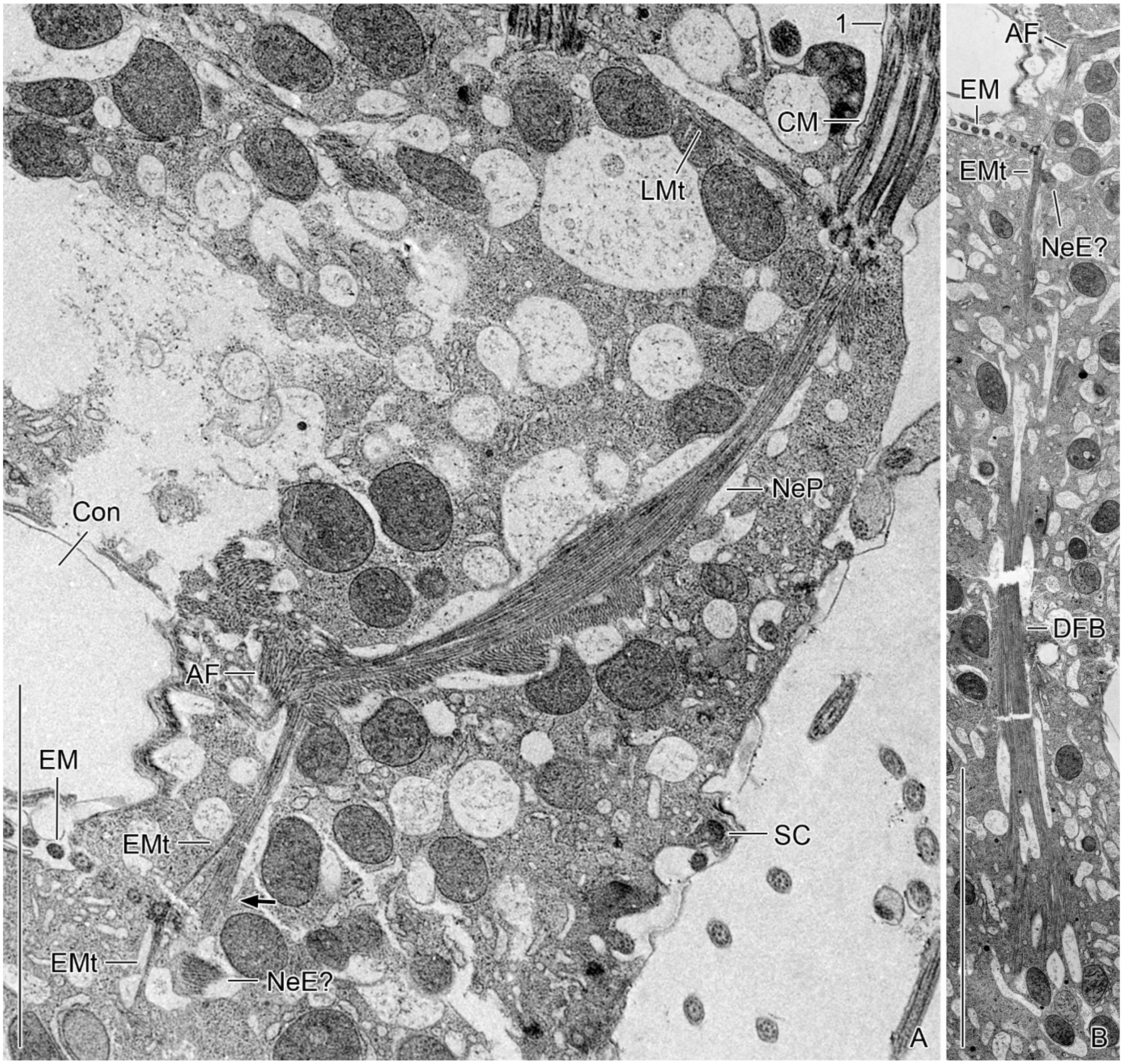
1
CM
LMt
NeP
Con
AF
EM
EMt
SC
EMt
NeE?
A
AF
EM
EMt
NeE?
DFB
B

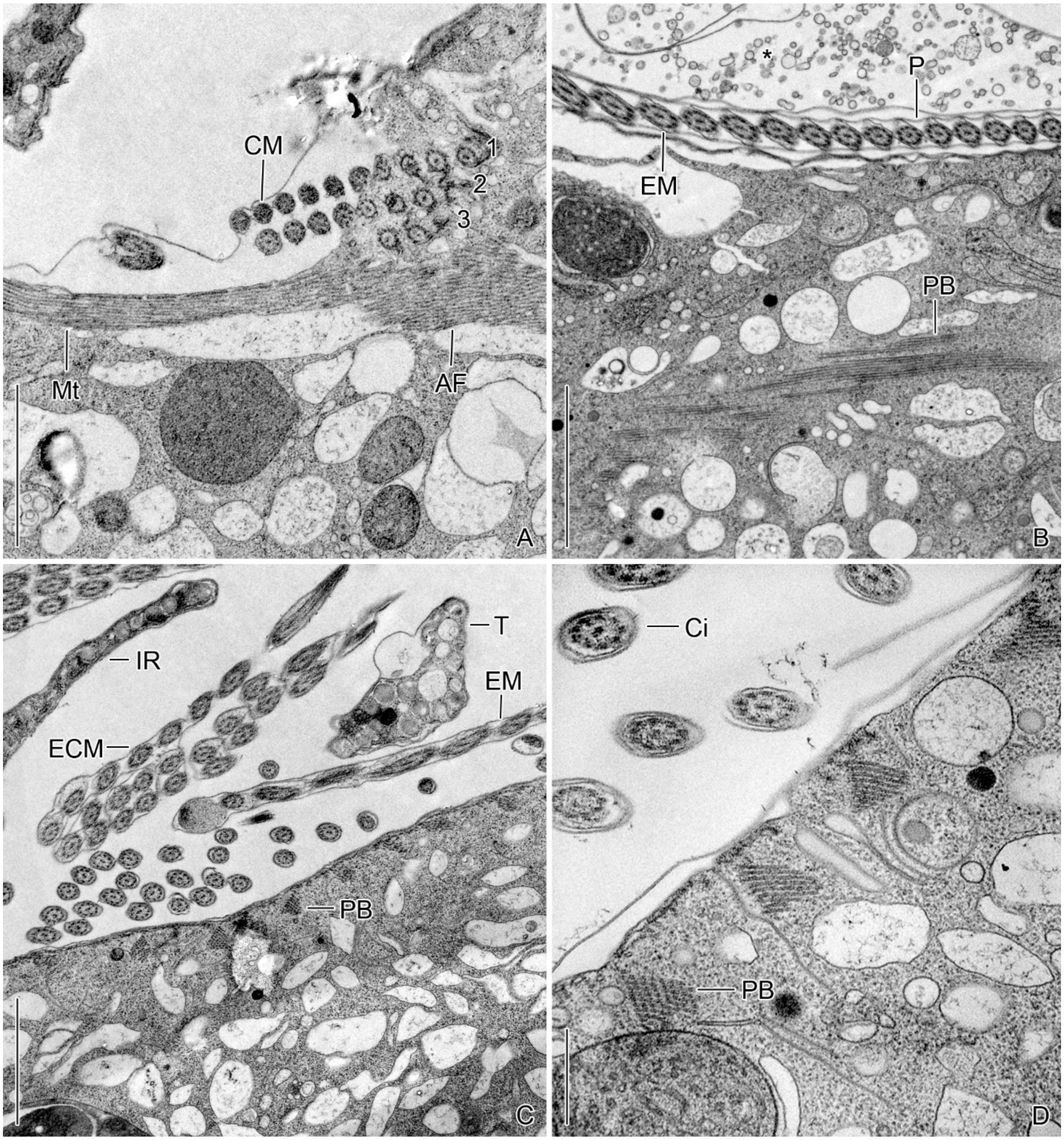
CM
1
2
3
Mt
AF
A
P
*
EM
PB
B
IR
T
EM
ECM
PB
C
Ci
PB
D

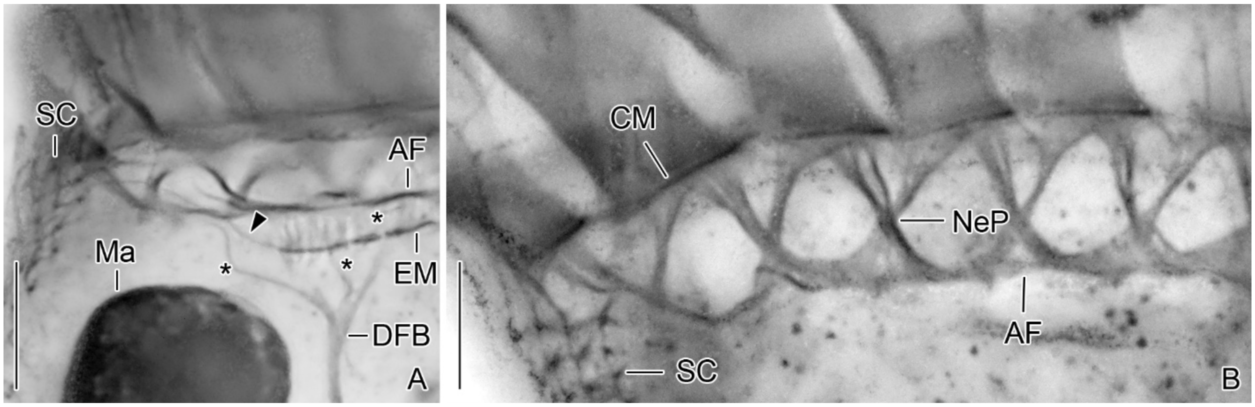
SC
AF
Ma
EM
DFB
A
CM
NeP
AF
SC
B

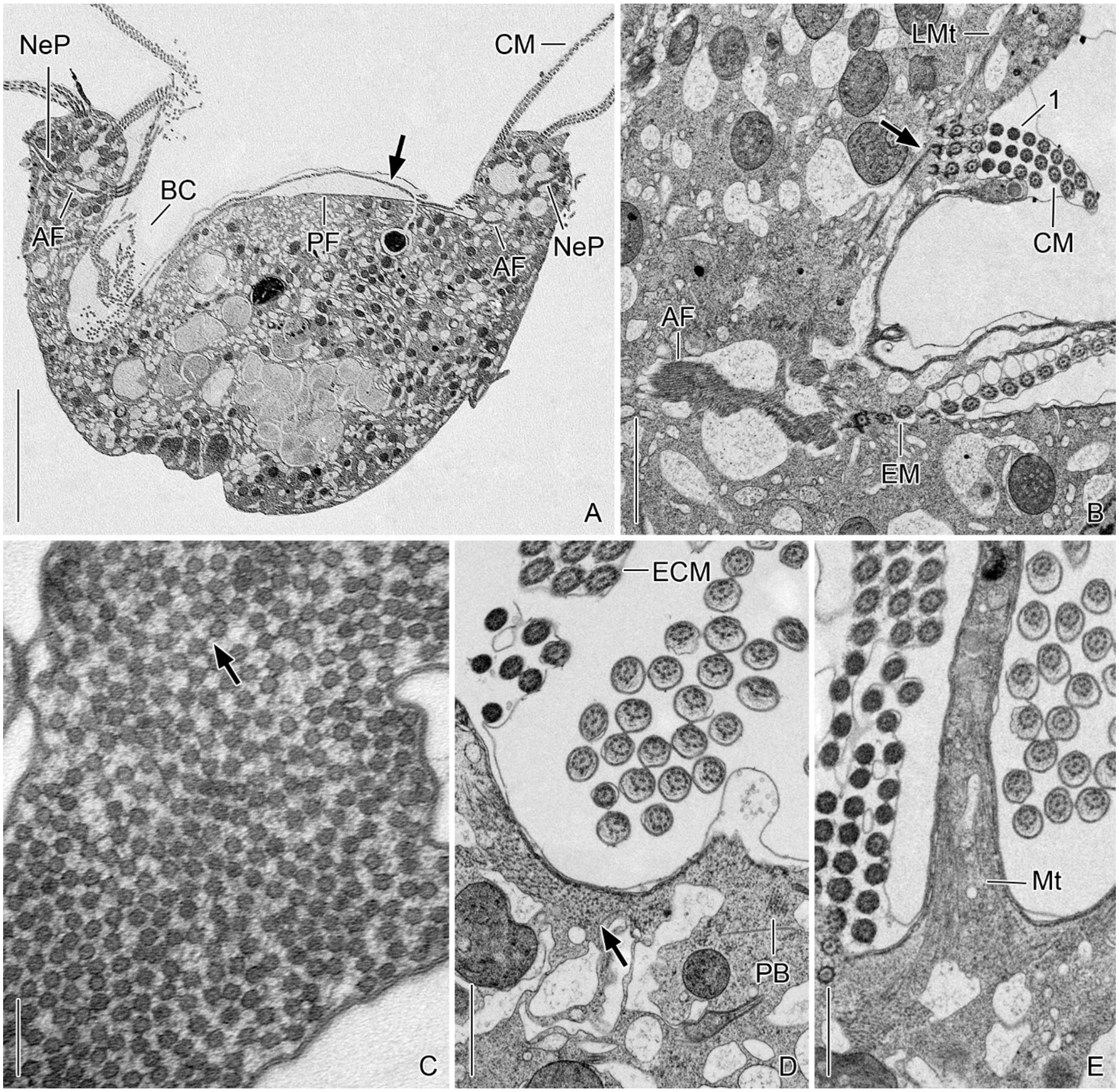
NeP
CM
BC
AF
PF
AF
NeP
A
LMt
1
CM
AF
EM
B
C
ECM
PB
D
Mt
E
